# Patient derived organoids to model rare prostate cancer phenotypes

**DOI:** 10.1038/s41467-018-04495-z

**Published:** 2018-06-19

**Authors:** Loredana Puca, Rohan Bareja, Davide Prandi, Reid Shaw, Matteo Benelli, Wouter R. Karthaus, Judy Hess, Michael Sigouros, Adam Donoghue, Myriam Kossai, Dong Gao, Joanna Cyrta, Verena Sailer, Aram Vosoughi, Chantal Pauli, Yelena Churakova, Cynthia Cheung, Lesa Dayal Deonarine, Terra J. McNary, Rachele Rosati, Scott T. Tagawa, David M. Nanus, Juan Miguel Mosquera, Charles L. Sawyers, Yu Chen, Giorgio Inghirami, Rema A. Rao, Carla Grandori, Olivier Elemento, Andrea Sboner, Francesca Demichelis, Mark A. Rubin, Himisha Beltran

**Affiliations:** 1 Department of Medicine, Division of Hematology and Medical Oncology, Weill Cornell Medicine, New York, NY 10021 USA; 2 Meyer Cancer Center, Weill Cornell Medicine, New York, NY 10021 USA; 3 Englander Institute for Precision Medicine,, Weill Cornell Medicine-New York Presbyterian Hospital, New York, NY 10021 USA; 4 Institute for Computational Biomedicine, Weill Cornell Medicine, New York, NY 10021 USA; 5 Center for Integrative Biology, University of Trento, 38123 Trento, Italy; 6 Cure First and SEngine Precision Medicine, Seattle, WA 98109 USA; 7 Human Oncology and Pathogenesis Program, Memorial Sloan Kettering Cancer Center, New York, NY 10065 USA; 8 Department of Pathology and Laboratory Medicine, Weill Cornell Medicine, New York, NY 10021 USA

## Abstract

A major hurdle in the study of rare tumors is a lack of existing preclinical models. Neuroendocrine prostate cancer is an uncommon and aggressive histologic variant of prostate cancer that may arise de novo or as a mechanism of treatment resistance in patients with pre-existing castration-resistant prostate cancer. There are few available models to study neuroendocrine prostate cancer. Here, we report the generation and characterization of tumor organoids derived from needle biopsies of metastatic lesions from four patients. We demonstrate genomic, transcriptomic, and epigenomic concordance between organoids and their corresponding patient tumors. We utilize these organoids to understand the biologic role of the epigenetic modifier EZH2 in driving molecular programs associated with neuroendocrine prostate cancer progression. High-throughput organoid drug screening nominated single agents and drug combinations suggesting repurposing opportunities. This proof of principle study represents a strategy for the study of rare cancer phenotypes.

## Abstract (web summary)

There are few available models to study neuroendocrine prostate cancer. Here they develop and characterize patient derived organoids from metastatic lesions, use these models to show the role of EZH2 in driving neuroendocrine phenotype, and perform high throughput organoid screening to identify therapeutic drug combinations.

## Introduction

Prostate cancer is the most common cancer in men and second leading cause of male cancer death in the United States^1^. Nearly all prostate cancer patients are diagnosed with prostate adenocarcinoma, which arises as an androgen-driven disease. Therefore, a highly effective therapeutic approach for patients with advanced disease is androgen deprivation therapy with gonadal suppression with or without the addition of chemotherapy or the potent androgen synthesis inhibitor abiraterone acetate^2,3^. However despite initial responses, castration resistance ultimately ensues. With recent therapeutic advances including more effective and earlier use of androgen receptor (AR)-targeted therapies, the landscape of castration-resistant prostate cancer (CRPC) is evolving^4^. While the majority of CRPC tumors remain AR-driven through the acquisition of activating AR mutations, amplification, splice variants, bypass, or other means, up to 10–20% of CRPC tumors lose AR dependence as a means to evade AR-targeted therapy^4^. One extreme manifestation is transformation from an AR-positive adenocarcinoma to an AR-negative small cell neuroendocrine carcinoma characterized by distinct morphologic features^5^. While small cell carcinoma of the prostate rarely arises de novo, castration-resistant small cell neuroendocrine prostate cancer evolves clonally from prostate adenocarcinoma during disease progression retaining early prostate cancer genomic alterations and acquiring distinct genomic, epigenetic, and pathway changes^6^. Patients with either de novo small cell neuroendocrine prostate cancer or castration-resistant neuroendocrine prostate cancer (CRPC-NE) are often treated with platinum-based chemotherapy similar to patients with small cell lung cancer; however, prognosis is poor and there are no known effective therapies beyond platinum.

While in vivo models have been described to model small cell neuroendocrine prostate cancer, the only widely available cell line is the NCI-H660 cell line, derived from a patient initially thought to have small cell lung cancer but later reclassified as prostate based on the presence of the prostate cancer-specific *TMPRSS2-ERG* gene fusion^7^. To expand on this unmet need, we developed patient-derived organoids from metastatic biopsies from patients with CRPC-NE. We molecularly characterized these new models and illustrate how they may be utilized to manipulate the expression and activity of oncogenes involved in the establishment of the neuroendocrine phenotype. High-throughput drug screening of patient-organoids nominated novel drug targets and combinations for CRPC-NE.

## Results

### Development of patient-derived tumor organoid and xenograft models

**Fig. 1:**
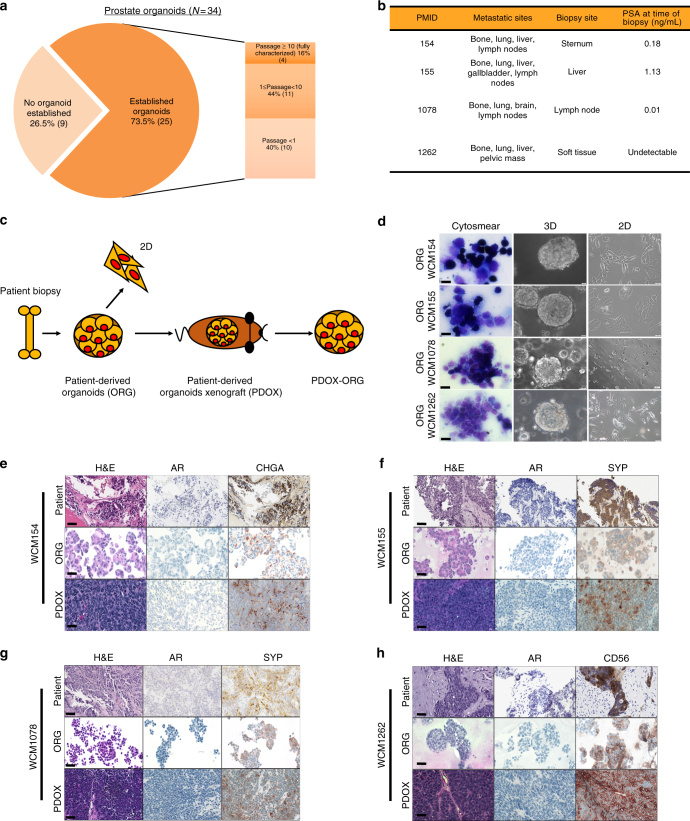
Development of patient-derived neuroendocrine prostate cancer models. **a** Pie chart of prostate cancer needle biopsies considered for the generation of organoids. No organoid established (light orange) represents no viable cells or no cellular material was found in culture after enzymatical digestion of the tissue. Established organoids (*p* < 1, lighter orange or 1 > *p* > 10 orange) refers to the presence of clusters of viable cells in culture that became senescent after few passages in culture. Passage >10, dark orange, indicates organoids that have been successfully grown in culture, molecularly characterized and used for functional studies and PDOX development. **b** Table of clinical data and biopsy sites of fully characterized CRPC-NE organoids. **c** Schematic view of the models generated from needle biopsies. In the scheme a patient biopsy is processed to generate 3D organoids (ORG). 3D organoids are then used to generate 2D cultures (2D) and also engrafted in an NSG mouse to grow patient-derived organoids xenograft (PDOX) and consequent organoids derived from the PDX (PDOX-ORG). **d** Air Dried Diff-Quick stained smears of organoids from small cell neuroendocrine prostate carcinoma and high grade adenocarcinomas with neuroendocrine features organoids (×40, scale bar 50 μm). Bright field image analysis (×40 magnification for 3D, ×20 magnification for 2D, scale bar 100 μm) of 3D and 2D organoids. **e**−**h** Histology images of native tumor biopsy tissue (Patient) compared with corresponding 3D organoid cultures (ORG), patient-derived organoids xenograft tissue PDOX (×20 magnification, scare bar 100 μm patient images, ×40, scale bar 50 μm for models). Samples are stained with hematoxylin-and-eosin (H&E) and immunohistochemistry (IHC) for AR, synaptophysin (SYP), chromogranin A (CHGA), and CD56

Fresh tumor tissue from 25 patients with metastatic prostate cancer was used for organoid development with an overall patient success rate of 16% (4/25) (Fig. 1a). Both three-dimensional (3D) and two-dimensional monolayer (2D) organoid-derived cell lines were successfully developed from four patients (liver, lymph node, soft tissue, and bone biopsy sites; Fig. 1b) and propagated (median 12 months) (Fig. 1c). During early passages, a cytology smear was performed to confirm the presence of tumor cells in the culture^8^ (Fig. 1d) and cancer-associated fibroblasts were isolated and propagated separately for further planned studies on the tumor microenvironment (Supplementary Fig.1). The organoids were also engrafted as patient-derived organoid xenografts (PDOXs) using NOD scid gamma (NOD.Cg-Prkdc^scid^ Il2rg^tm1Wjl^/SzJ) mice and subsequently re-passaged in vitro as organoids from PDOXs (PDOX-ORG) (Supplementary Fig. 2).

The pathology of each of the four patient’s metastatic tumor and their matched organoids and PDOXs was classified as neuroendocrine prostate cancer based on tumor morphology, including both pure small cell carcinoma and high-grade carcinoma with extensive neuroendocrine differentiation, and were characterized by the presence of small- to medium-sized round cells with fine chromatin pattern and nuclear molding^9,10^. All four organoids and their PDOXs lacked AR protein expression and expressed classical neuroendocrine markers by immunohistochemistry (Fig. 1e−h).

### Molecular characterization of neuroendocrine models

**Fig. 2:**
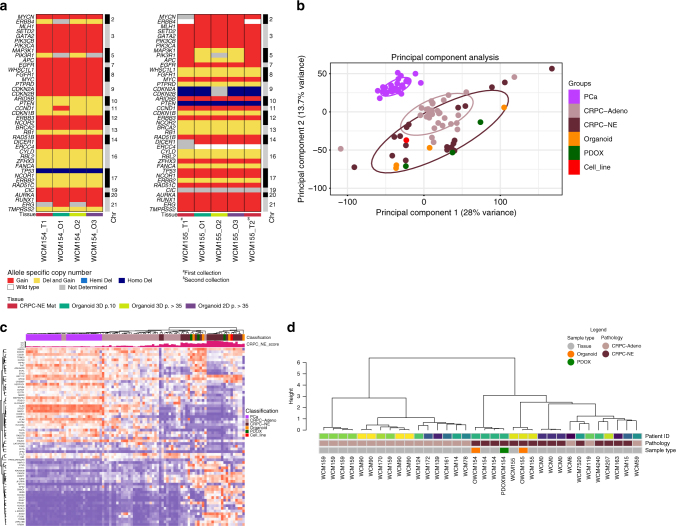
Molecular characterization of patient-derived neuroendocrine prostate cancer models. **a** Allele-specific copy number of selected genes in organoids at different passages over time. **b** Transcriptomic analysis via principle component analysis including 26 PCA, 33 CRPC-Adeno, 13 CRPC-NE patient samples and CRPC-NE organoids (orange), CRPC-NE PDOX (green), NIC-H660 cell line (red) using RNAseq expression (FPKMS) of ~20k genes. **c** Clustering of study cohort samples using genes involved in CRPC-NE phenotype, based on RNAseq Expression (FPKMS). The cohort of localized prostate adenocarcinoma PCA (pink), CPRC-Adeno (mauve), and CRPC-NE (brown) patients (cohort from Beltran et al.^6^) CRPC-NE organoids (orange), CRPC-NE PDOX (green), NIC-H660 cell line (red). The green barplot on the top of the heatmap represents the CRPC-NE score (range from 0, low to 1, high) calculated according to methodologies described in Beltran et al.^6^. **d** Genome-wide DNA methylation cluster analysis using a cohort of CPRC-Adeno (mauve) and CRPC-NE (brown) patients^6^ together with models generated (organoids in orange and PDOX in green)

To determine how genomically stable the organoids are, we performed whole-exome sequencing (WES) of organoids in both 2D and 3D culture conditions and at serial timepoints (passages 10 and 35) and compared these results with the patient’s matched metastatic tumor biopsy and PDOX. Tumor purity of all models including organoids, PDOXs, and PDOX-derived organoids estimated by CLONET^11^ was high (median 98%) (Supplementary Fig. 3). Genome-wide copy number alterations were concordant across models and with time including genes commonly altered in advanced prostate cancer^6,12^ (Fig. 2a, Supplementary Fig. 4).

Using RNA-seq and principal component analysis, we compared the patient organoids and matched PDOX transcriptome profiles with a published cohort of 26 localized prostate adenocarcinoma (PCA), 33 metastatic castration-resistant adenocarcinoma (CRPC-Adeno), and 13 CRPC-NE patient tumors, and found consistent segregation of the CRPC-NE organoids with CRPC-NE patient tumors (Fig. 2b). The organoids and PDOXs clustered based on their shared expression of CRPC-NE signature genes^6^ (Fig. 2c) including overexpression of *MYCN*^13^, *PEG10*^14^, *SRRM4*^15^, *EZH2*^6^, *SOX2*^16^, *BRN2*^17^, and *FOXA2*^2^ (Supplementary Fig. 5), and low expression of AR signaling genes^18^ (Supplementary Fig. 6). There were no significant differences in gene expression between 2D and 3D cultures (correlation coefficient 0.934, Supplementary Fig. 7) or in media with or without DHT (Supplementary Fig. 8).

Although the retinoblastoma gene *RB1*, commonly deleted in CRPC-NE and other small cell carcinomas, was not lost at the genomic level in any of our CRPC-NE organoids, transcriptome analysis revealed pathway dysregulation consistent with RB1 loss^19^. This suggests a loss of function of the RB1 pathway by other means, which we found in these cases due to aberrant phosphorylation of RB1 and/or inactivation/deletion of CDK-inhibitor p16ink4a (CDKN2A) **(**Supplementary Fig. 9), mechanisms previously described^20,21^.

Based on the marked epigenomic changes previously reported in CRPC-NE patient biopsies^6^, we also evaluated CpG-rich methylation in organoids on a genome-wide scale using enhanced reduced representation bisulfite sequencing (ERRBS). The patient-derived organoid models clustered with their corresponding patient tumors based on DNA methylation as well as with other CRPC-NE cases using our published datasets (Fig. 2d).

Based on the presence of genomic alterations involving common cancer-associated genes (Supplementary Fig. 10) and mRNA and DNA methylation clustering with patient tumors of the same disease state, the models appeared representative of their matched patient and of CRPC-NE. The high tumor purity of the models, consistent IHC analysis of common markers across tumor cells, and lack of expression of benign markers (i.e., benign liver marker Hep Par 1 in the liver biopsy-derived organoid (Supplementary Fig. 11)) suggested limited cellular heterogeneity and supported a lack of contamination by benign tissues but also the inability to maintain features of the microenvironment or multiple tumor populations with time.

### Effects of EZH2 inhibition

**Fig. 3:**
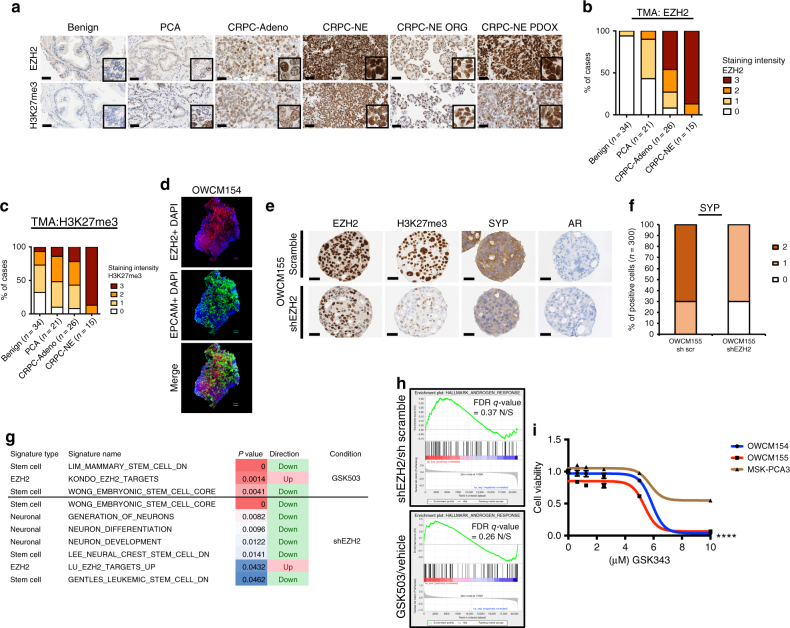
Manipulation of EZH2 in CRPC-NE models affects neuroendocrine-associated programs and tumor cell viability. **a** Representative EZH2 IHC images of benign prostate, localized prostate cancer (PCA), CRPC-Adeno and CRPC-NE patient tissue, representative CRPC-NE organoids, and corresponding PDOX. Tissue is stained with EZH2 and H3K27me3 antibodies (×20 magnification, scale bar 100 μm, inset ×40). **b** Bar plots scoring analysis of tissue staining intensity using EZH2 and H3K27me3 antibodies. The cases are represented as % and the number of cases is indicated in the figure. EZH2 and H3K27me3 staining intensity vary from 0 (no to minimal intensity) to 4 (high intensity). **c** Immunofluorescence staining of OWCM154 using EZH2 (Alexa Fluor^®^ 555) and APC anti-human EPCAM and 4′,6-diamidino-2-phenylindole, dihydrochloride (DAPI) (scale bar 30 μm). **d** Immunohistochemistry of OWCM155 organoids infected with sh scramble or shEZH2. Organoids are stained using EZH2, H3K27me3, synaptophysin (SYP), and AR antibodies. (×40 objective, scale bar 50 μm). **e** Bar plot scoring analysis of SYP staining intensity in shEZH2 versus sh scramble. Staining intensity is calculated from 0 (no to minimal intensity) to 2 (medium-high intensity). Three hundred cells have been evaluated for the scoring. **f** GSEA table of signatures (EZH2 targets, Neuronal and Stem Cell) that are significantly enriched in organoids treated with shEZH2 compared to scramble and in organoids treated with GSK503 compared to vehicle treatment. These signatures are ranked based on *p* value (from 0.05 to 0.001) and FDR < 0.25. **g** GSEA enrichment plot of the AR pathway genes in organoids infected with shEZH2 versus scramble or treated with EZH2 inhibitor (GSK503) or vehicle. **h** Cell viability assay (Cell-Title Glo) of OWCM154 (blue), OWCM155 (red), MSK-PCA3 (dark orange) after 11 days of treatment with vehicle or indicated doses of GSK343 (*n* = 9, for each treatment dose, error bars: s.e.m.) Two-way ANOVA test is used, ****p < 0.0001 (OWCM154 vs MSK-PCA3 and OWCM155 vs MSK-PCA3)

The histone methyltransferase enhancer of zeste 2 (EZH2) is an epigenetic modifier frequently overexpressed in many cancer types including prostate cancer and supports cancer cell proliferation and survival^22–25^. Recent work by our group and others has identified EZH2 as a potential mediator of CRPC-NE progression^6,13,26–28^. We evaluated a larger cohort of patients and identified overexpression of EZH2 protein expression in the majority (87%) of CRPC-NE tumors (*n* = 15) compared with 46% of CRPC-Adeno (*n* = 26), 5% localized prostate adenocarcinoma (*n* = 21), and minimal to no expression in benign prostate tissues (0%, *n* = 34). Overexpression of EZH2 was associated with concomitant increased EZH2 activity (i.e., H3K27me3 expression). The levels of EZH2 and H3K27me3 were comparable in CRPC-NE organoid and PDOX models (Fig. 3a, b and Supplementary Fig. 12) and expression of EZH2 in the nuclei was visualized using immunofluorescence (Fig. 3c). The cell cycle regulator E2F1 positively controls EZH2 transcription^29^. As expected, the expression of EZH2 and E2F1 in our patient cohorts and organoids was highly correlated (*r* = 0.879, *p* value < 2.2e-16) (Supplementary Fig. 13).

To gain further insights into how epigenetic modulation might affect neuroendocrine prostate cancer progression, we successfully infected human CRPC-NE organoids with short hairpin RNA targeting EZH2 or a scramble sequence. Knockdown of EZH2 resulted in a reduction of its activity measured through the H3K27 methylation and a decrease in expression of classical neuroendocrine markers including synaptophysin (SYP) but remained AR-negative (Fig. 3d, e). By gene set enrichment analysis (GSEA), we found a significant upregulation of EZH2-suppressed target genes and downregulation of stem cell and neuronal programs after knockdown (Fig. 3f). EZH2 has been associated with stem cell properties and tumor-initiating cell function in different cancer types including glioblastoma, breast, and pancreatic cancers^24,30^. GSEA of organoids treated with the EZH2 inhibitor GSK503 demonstrated similar results as with shRNA (Fig. 3f, Supplementary Fig. 14 and Supplementary Data1), though neuronal pathways did not reach statistical significance (*p* value < 0.05 and FDR < 0.25). Again, no upregulation was observed in AR expression or AR signaling by interfering with EZH2 activity (Fig. 3g). Taken together, these data suggest that EZH2 is associated with CRPC-NE program dysregulation, but suppression of EZH2 alone is not sufficient to re-express AR in this late-stage AR-negative CRPC-NE state. This differs from what has been recently described in other prostate cancer models of lineage plasticity in which EZH2 inhibition resulted in re-expression of the AR^26,28^, possibly due to an earlier more “plastic” disease state in those models where AR was not completely absent prior to therapy.

To understand whether inhibition of EZH2 activity could be considered as a treatment option for CRPC-NE, we treated CRPC-NE organoids with the EZH2 inhibitors GSK343 and GSK503. This resulted in a reduction of H3K27me3 expression (Supplementary Fig. 15) and a preferential decrease in the viability in CRPC-NE organoids at high doses compared to CRPC-Adeno organoids used as control^31^ (Fig. 3h, Supplementary Fig. 16). These results were confirmed measuring cell death by annexin staining (Supplementary Fig.17). To reinforce the terminal differentiation hypothesis, when we treated CRPC-NE organoids with an EZH2 inhibitor in combination with the AR antagonist enzalutamide, no additive effects or synergy were observed (Supplementary Fig. 18). These data suggest that EZH2 inhibition has activity in CRPC-NE and this does not require expression of the AR. However given the high doses required, combination therapies may be required similar to what has been described in other cancer types^26,32,33^.

### High-throughput drug screening

Given a lack of therapeutic options available for patients with CRPC-NE and our observed EZH2 inhibitor single agent activity, we explored the activity of existing drugs and drug combinations by performing a high-throughput drug dose−response screen using a drug library of 129 chemotherapeutics and targeted agents^34^. We tested the four CRPC-NE organoids as well as two CRPC-Adeno organoids as controls^31^.

**Fig. 4:**
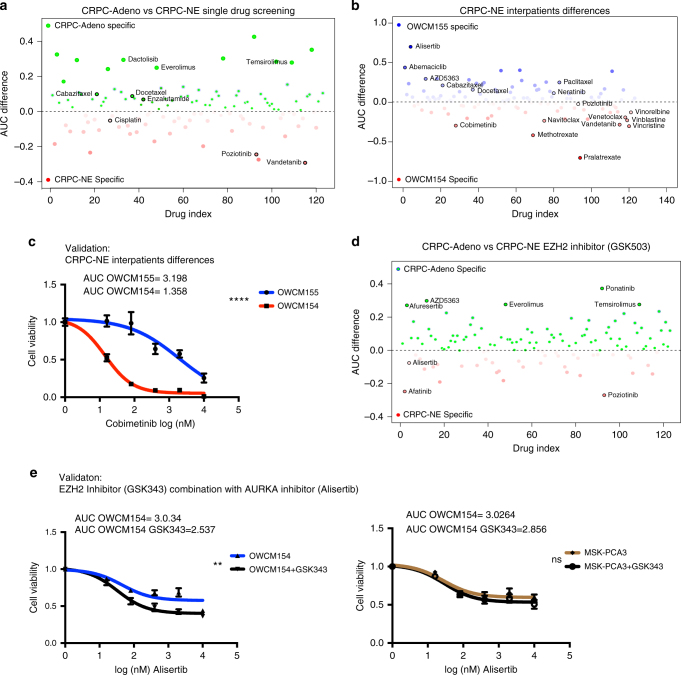
High-throughput drug screening in organoids identifies novel single agents and combination therapies for CRPC-NE. **a** High-throughput drug screen in CRPC-NE organoids (OWCM154 OWCM155) vs control CRPC-Adeno organoids (MSK-PCA3 MSK-PCA2). The *y-*axis is the AUC (area under the curve) differential of the mean of the CRPC-NE samples – the mean of the CRPC-Adeno samples. Compounds indicated in red are specific for CRPC-NE while compounds indicated in blue are specific for CRPC-Adeno. Highlighted compounds are clinically relevant and represent a subclass of drugs. **b** High-throughput drug screen single agent analysis of differences in sensitivity within the CRPC-NE samples. **c** Cell viability assay using vehicle or different doses of cobimetinib in OWCM154 and OWCM155 (*n* = 9, for each treatment dose, error bars: s.e.m.), two-way ANOVA test is used. ****p < 0.0001. **d** High-throughput drug combination screen in CRPC-NE organoids (OWCM154, OWCM155) vs CRPC-Adeno organoids (MSK-PCA3, MSK-PCA2). GSK503 has been added to the drug screening plate at IC30. The *y-*axis is the AUC differential of the mean of the CRPC-NE samples – the mean of the CRPC-Adeno samples. Compounds indicated in red are more effective for CRPC-NE in combination with GSK503 while compounds indicated in blue are more effective for CRPC-Adeno in combination with GSK503. **e** Cell viability assay for high-throughput drug combination screen validation. Organoids are treated with a fixed dose of GSK343 (5 μM) and escalated doses of alisertib. The organoids treated with alisertib in combination with GSK343 are represented with a black line while the organoids treated with alisertib plus DMSO are represented in blue (*n* = 9, for each treatment dose, error bars, s.e.m.). Two-way ANOVA test is used. Alisertib-GSK503 combination in OWCM154 has *p* < 0.00.27, (**) while in MSK-PCA3 is not significant (ns)

As expected, drugs approved for patients with CRPC-Adeno including enzalutamide, an AR-antagonist, and the taxane chemotherapies cabazitaxel and docetaxel^35,36^ were identified as active in CRPC-Adeno organoids based on the drug screen. The drug screening results for CRPC-NE vs CPRC-Adeno organoids nominated a modest number of drugs such as pozotinib (HER) and vandetanib (VEGFR2) more effective in killing CRPC-NE over control CRPC-Adeno tumor cells (Fig. 4a, Supplementary Fig. 19).

High-throughput drug screening also highlighted patient-specific sensitivities (Fig. 4b). For instance, the CRPC-NE organoid OWCM155 exhibited significant sensitivity to the aurora kinase inhibitor alisertib consistent with the corresponding patient’s exceptional response in the Phase 2 trial of alisertib for CRPC-NE (NCT01482962) (Beltran et al. ESMO)^37^. We confirmed responses to single agents including alisertib and GSK343 in vitro using both cancer and benign prostate cell line and organoids (Supplementary Fig. 20). CRPC-NE organoid OWCM1078 similarly responded well to alisertib (Supplementary Fig. 21**)**. On the other hand, the CRPC-NE organoid OWCM154 did not respond to alisertib in vitro (nor did the patient on the phase 2 clinical trial) but demonstrated response to the MEK inhibitor cobimetinib (Fig. 4c). These data support a potential role of organoid drug screening to predict individual patient responses to therapy. Drug screening also identified drugs predicted by genomic alterations. For instance OWCM155, which harbored *PTEN* deletion by WES and had high basal levels of phospho-AKT, was particularly sensitive to AKT inhibition (AZD5363, afuresertib) (Supplementary Fig. 22).

The CRPC-NE and CRPC-Adeno organoids were tested in a drug combination screen adding sub-lethal doses of the EZH2 inhibitor, GSK503. For OWCM154, one of the top GSK503 combinations that enhanced the effect of the single agent was with alisertib (AURKA) (Fig. 4d, e and Supplementary Fig. 23). As described the OWCM154 organoid (and corresponding patient) was resistant to alisertib as single agent; these data suggest that targeting two pathways implicated as cooperators for CRPC-NE progression^13^ may be an effective approach and may be picked up through an unbiased screen. For the alisertib responder organoids, other drugs nominated as effective in combination with GSK503 included the EGFR class of inhibitors (neratinib, afatinib, erlotinib, and osimertinib). The CRPC-Adeno organoids did not show increased sensitivity to these combinations.

## Discussion

While there have been significant advances in the treatment of patients with advanced prostate cancer, there is a wide variability in clinical responses to existing drugs. There are few preclinical models that recapitulate the clinical and molecular heterogeneity seen among patients thereby limiting the rational development of molecularly driven treatment strategies. Here we focus on the CRPC-NE phenotype, an emerging and aggressive subtype of advanced prostate cancer that can arise as an androgen-independent mechanism of resistance to AR-directed therapies, due to the lack of approved therapies for patients, limited preclinical models (only one cell line is available through ATCC), and a still preliminary understanding of CRPC-NE biology.

As with other rare cancers, there are few drugs or trials that have been developed for patients with CRPC-NE. Here we show that gaps in our knowledge concerning rare cancers may be addressed through the development of patient-derived preclinical models. Patient organoids retain the molecular features of their corresponding patient over time and maintain similar responses to drugs in vitro.

Attempts to create prostate cancer organoids from biopsies have also been performed by other laboratories with similarly low overall success rates for indefinite propagation and expansion, perhaps due to the inability of cells to adapt very quickly from tissue to the culture conditions and therefore avoiding senescence. The development of models from biopsies also faces the challenge of scant starting material derived from needle biopsies of metastatic sites (especially bone), limiting the cell−cell interactions that are needed for cells to survive. Of the seven prostate cancer organoids described in Gao et al., one was from a patient with CRPC-NE derived from a malignant pleural effusion^31^. Other systems for organoid expansion have been established as using irradiated mouse cells and a Rho kinase (ROCK) inhibitor as supportive elements for human epithelial cells^38^ or a matrigel/EGF-based culture system supplemented with androgens^39^. It has been shown that higher cell density deactivates mTOR pathway and suppress the senescence program^40^. The use of ROCK inhibitors while passaging organoids delays senescence and supports proliferation programs^41,42^ but tissue processing and media optimization are required to make this more suitable for low biopsy input cellular amount. Further, the optimal media conditions to support CRPC-NE vs CRPC-Adeno may be different and whether co-culturing techniques may improve success rates are yet to be elucidated.

Previously described patient-derived xenograft (PDX) models of neuroendocrine prostate cancer have been generated from larger quantities of surgical or autopsy material^43^ including LuCaP 49^44^, MDA PCA 144 PDX^45^, LTL352 and LTL370^46^, LTL545^47^ and these represent complementary clinically relevant models to study CRPC-NE biology and therapeutic strategies. Adding the organoid development step from smaller input material including needle biopsies could positively impact the ability to generate patient in vivo models.

We used organoids to assess the functional impact of genes involved in CRPC-NE pathogenesis and highlight the role of EZH2. EZH2 inhibition resulted in a downregulation of neuroendocrine pathway genes and those associated with stem cell and neuronal pathways; however, AR expression or activity did not increase suggesting a later disease state and possibly loss of plasticity and inability of these CRPC-NE organoids to revert back to a more luminal state.

Organoid drug screening generated hypotheses for single-agent and combination therapies confirming the usage of certain drugs in the clinic for CRPC-Adeno and nominating new approaches for CRPC-NE. There are currently no approved therapies for CRPC-NE, representing a clinical unmet need. We previously reported preferential sensitivity of CRPC-NE to aurora kinase A inhibition, which led to a multicenter phase 2 trial of alisertib for CRPC-NE (NCT01482962). The organoid models OWCM155 and OWCM154 were developed from an exceptional responder and non-responder patient enrolled on the phase 2 clinical trial and demonstrated corresponding responses to alisertib in vitro. Drug screening also identified drugs and drug combinations concordant with the genomic background of the tumors, as was the case of *PTEN* loss that conferred response to AKT inhibition. Combination screens using an EZH2 inhibitor as a potential method for priming to other treatments identified novel combinations not yet tested in the clinic for CRPC-NE patients. For instance, EZH2 inhibition combined with the AURKA inhibitor alisertib, both tested as single agents in CRPC-NE, were identified possibly due to their cooperative role in driving N-myc activity in CRPC-NE^20^. Although additional studies are needed to further understand the biologic implications of several of these findings, these data suggest that CRPC-NE organoids are clinically relevant models to unveil novel targets and therapies, and high-throughput drug screening is a useful tool to generate valid treatment hypotheses for CRPC-NE.

## Methods

### Cohort description and pathology classification

Fresh tumor biopsy specimens were obtained prospectively through a clinical trial approved by the Weill Cornell Medicine (WCM) Institutional Review Board (IRB) with informed consent (IRB #1305013903). Germline (normal) DNA was obtained from peripheral blood mononuclear cells. All hematoxylin and eosin-stained slides were reviewed by board-certified pathologists (J.M.M. and M.A.R.). Histologic criteria were from the proposed classification of prostate cancer with neuroendocrine differentiation^9^.

### Tissue processing and organoid development

Fresh tissue biopsy samples were placed in media DMEM (Invitrogen) with GlutaMAX (1×, Invitrogen), 100 U/ml penicillin, 100 μg/ml streptomycin (Gibco), Primocin 100 μg/ml (InvivoGen), and 10 μmol/l ROCK inhibitor (Selleck Chemical Inc.). Tissue samples were washed in media two times before being placed in a 10 cm petri dish (Falcon) for mechanical dissection. The dissected tissue was then enzymatically digested with 250 U/ml of collagenase IV (Life Technologies) and TrypLE express (Gibco) in a ratio 1:2 with Collagenase IV in a 15 ml conical centrifuge tube (Falcon) incubated in a shaker at 37 °C set to 5 rcf. Incubation time of the specimen was dependent on the amount of collected tissue and ranged from 30 to 90 min, until the majority cell clusters were in suspension. After tissue digestion, DMEM media containing 10% FBS was added to the suspension to inactivate collagenase IV and the mixture was centrifuged at 326 rcf for 4 min. The pellet was then washed with Advanced DMEM (Invitrogen) containing GlutaMAX (1×, Invitrogen), 100 U/ml penicillin, 100 μg/ml streptomycin (Gibco), and HEPES (1 M, Gibco). The pellet was resuspended with prostate-specific culture media composed of Advanced DMEM (Invitrogen) with GlutaMAX (1×, Invitrogen), 100 U/ml penicillin, 100 μg/ml streptomycin (Gibco), Primocin 100 μg/mL (InvitroGen), B27 (Gibco), *N*-Acetylcysteine 1.25 mM (Sigma-Aldrich), Mouse Recombinant EGF 50 ng/ml (Invitrogen), Human Recombinant FGF-10 20 ng/ml (Peprotech), Recombinant Human FGF-basic 1 ng/ml (Peprotech), A-83-01 500 nM (Tocris), SB202190 10 μM (Sigma-Aldrich), Nicotinaminde 10 mM (Sigma-Aldrich), (DiHydro) Testosterone 1 nM (Sigma-Aldrich), PGE2 1 μM (R&D Systems), Noggin conditioned media (5%) and R-spondin conditioned media (5%). The final resuspended pellet was combined with growth factor-reduced Matrigel (Corning) in a 1:2 volume ratio. Six droplets of 50 μl cell suspension/Matrigel mixture was pipetted onto each well of a six-well cell suspension culture plate (Sarstedt Ltd.). The plate was placed into a cell culture incubator at 37 °C and 5% CO_2_ for 30 min to solidify the droplets before 3 ml of prostate-specific culture media was added to each well. The culture was replenished with fresh media every 3−4 days during organoid growth. Dense cultures with organoids ranging in size from 200 to 500 um were passaged weekly. During passaging, the organoid droplets were mixed with TrypLE Express (Gibco) and placed in a water bath at 37 °C for a maximum of 5 min. The resulting cell clusters and single cells were washed and replated, following the protocol listed above. Prostate organoids were biobanked using Recovery Cell Culture Freezing Medium (Gibco) at −80 °C. Throughout prostate organoid development, cultures were screened for various *Mycoplasma* strains using the MycoAlert Kit (Lonza) and confirmed negative before being used for experimental assays. The MSK-PCA2 and MSK-PCA3 used for this study as CRPC-Adeno controls were developed and described by Gao et al.^31^.

### Patient-derived organoid xenograft development

1.5 million cells derived from organoids were injected with Matrigel (Corning) 1:1 subcutaneously into NOD scid gamma (NOD.Cg-Prkdc^scid^ Il2rg^tm1Wjl^/SzJ) male mice (Jackson Laboratories, Bar Harbor, Maine). Mice used for xenografts were 6-to 8-weeks old. Daily light cycles were kept consistent in the animal facility (12 h light and 12 h dark). Cages were changed fully once a week. Tumor volume was measured every week with a caliper. The animals were sacrificed in a CO_2_ chamber after 2−4 months of tumor growth. The harvested tumors were partly used for histology, genomic and transcriptomic analysis and partly rengrafted into NOD scid gamma mice. Animal care and experiments were carried out in accordance with IACUC guidelines.

### Immunoblot and immunohistochemistry and immunofluorescence

Organoids were lysed in RIPA buffer supplemented with protease inhibitor cocktail and phosphatase inhibitors (Thermo Scientific). In the case of H3K27me3 detection sonication was performed (High, 30″ on and 30″ off for 5 cycles). The total protein concentration of the soluble extract was determined using the BCA protein assay Kit (Thermo Scientific). Each protein sample (50 μg) was resolved to SDS-PAGE, transferred onto a PVDF membrane (Millipore) and incubated overnight at 4 °C with primary antibodies. Primary antibodies used: Androgen Receptor (1:2000, Abcam, [ER179(2)] ab108341), PTEN (D4.3) XP (1:1000, Cell Signaling 9188S), Actin (1:2000, EMD Millipore clone C4, MAB1501), H3K27me3 (1:1000, Cell Signaling Technology, 9733S), EZH2 (D2C9) XP^®^ Rabbit mAb (1:1000, Cell Signaling 5246). Phospho-Rb (Ser780) (1:1000, Cell Signaling, 9307), CDKN2A (1:2000, Abcam, ab108349), Synaptophysin (1:5000, Abcam [YE269], ab32127). Following three washes with TBS-T, the blot was incubated with horseradish peroxidase-conjugated secondary antibody and immune complexes were visualized by enhanced chemiluminescence detection (ECL plus kit, Pierce).

Immunohistochemistry was performed on deparaffinized FFPE sections (organoid, xenograft or patient tissue) using a Bond III automated immunostainer (Leica Microsystems, IL, USA). Heat-mediated antigen retrieval was performed using the Bond Epitope Retrieval solution 1 (ER1) at pH6 or 2 (ER2) at pH9. The following antibodies and conditions were used: EZH2 (clone 11/EZH2, BD Biosciences, CA, USA; ER1, 1:20 dilution), H3K27me3 (C36B11, Cell Signaling, MA, USA; ER1, 1:200 dilution), Synaptophysin (SP11, Thermo Scientific; ER2, 1:100 dilution), Chromogranin A (LK2H10, BioGenex, CA, USA; no antigen retrieval, 1:400 dilution), AR (F39.4.1, BioGenex, CA, USA; ER1, 1:800 dilution with casein), Ki67 (MIB-1, Dako, CA, USA; ER1, 1:50 dilution), PTEN (Cell Signaling, 9559, ER2, 1:100).

Scoring of EZH2 and H3K27me3 was performed on tissue microarrays (85 cases) and whole slides (11 cases). Nuclear staining intensity in tumor tissue was evaluated blindly by a pathologist using a four-tiered scoring system: negative (or present in <5% of tumor nuclei), weak, moderate or strong. If a case showed heterogeneous staining, the intensity score representative of the majority of tumor nuclei of that case was assigned.

Scoring of Synaptophysin was performed blindly by a pathologist analyzing 300 cells on slides sh scramble vs shEZH2 and applying the following scoring system: 0 negative staining, 1 weak, 2 mild staining.

Immunofluorescence was performed on OWCM154 and OWCM155 using the following antibodies EZH2 (D2C9) XP^®^ Rabbit mAb (1:250, Cell Signaling 5246), secondary antibody Alexa Fluor^®^ 555 (1:1000, ThermoFisher Scientific A27039) and APC anti-human EPCAM (1:250, Biolegend 324208) DAPI. Briefly, organoids were washed with PBS and Paraformaldehyde (PFA 4% in PBS) was added overnight at 4°C. The following day organoids cells were incubated with a blocking solution containing 1% Triton™-X and 1% FBS in PBS for 60 min at room temperature. Primary and secondary antibodies were added in PBS solution containing 0.5% Triton™-X and 0.1% FBS for 1 h respectively at indicated concentration at room temperature^48^. Z-stacks are obtained using a Zeiss confocal microscope (LSM510; Carl Zeiss Microscopy). 3D images are obtained combining the Z-stacks using Imaris software.

### Cytology smear

Organoids at early passage were morphologically screened for contamination of benign epithelial cells and fibroblasts. Organoids cells were collected from the Matrigel droplet using an inverted microscope and placed on Super Frost PLis glass slide (VWR MicroSlides # 48311-703). A second glass slide was used to spread the organoids on the entire surface and after air-drying stained with Diff-Quik stain (Siemens Medical Solution USA, INC, Mavren Pa). The stained organoids were reviewed by the study pathologists.

### DNA extraction and exome sequencing

DNA extractions from patient tumors, organoids, and PDOXs were performed using DNeasy Blood and Tissue Kit (QIAGEN) and Maxwell 16 Tissue DNA Purification Kit (Promega). Whole-exome capture libraries were constructed after sample-shearing, end repair, and phosphorylation and ligation to barcoded sequencing adaptors. Ligated DNA was size-selected for lengths between 200 and 350 bp and subjected to HaloPlex Exome (Agilent). Sequencing was performed using Illumina HiSeq 2500 (2 × 100 bp). Reads were aligned to GRC37/hg19 reference using Burrows-Wheeler Aligner and processed according to the IPM-Exome-pipeline v0.9.

### Copy-number analysis

Concordance between tumor tissues, tumor organoid models, and matching xenografts was assessed using SPIA^49^ genotype distance test. CLONET^11^ was used to quantify tumor purity and ploidy from WES segmented data and allelic fraction (AF) of germline heterozygous SNP loci. A pair (cnA, cnB) of integer values, representing allele-specific copy number, was assigned to each genomic segment identified by the IPM-Exome-pipeline, as described in Beltran et al.^6^. Quality filters required at least ten informative SNPs and mean coverage of 20 to call allele-specific values of a segment. Post-processing manual review of allele-specific calls was performed.

Concordance between two tumor samples was assessed by comparing discretized allele-specific copy number values into five levels (Fig. 2a): homozygous deletion (cnA = 0, cnB = 0), hemizygous deletion (cnA = 1, cnB = 0), wild type (cnA = 1, cnB = 1), gain (cnA ≥ 2, cnB ≥ 1), and reciprocal loss of heterozygosity (cnA > 1, cnB = 0). Reciprocal loss of heterozygosity event captures complex copy number states where one allele is lost, and the other one is gained. Reciprocal loss of heterozygosity was conserved in tumor organoids models and matching xenografts.

### RNA extraction sequencing and analysis

mRNA was extracted from organoids and PDXs using RNAsy Mini Kit (QIAGEN) and Maxwell 16 LEV simplyRNA Tissue Kit. Specimens were prepared for RNA sequencing using TruSeq RNA Library Preparation Kit v2 as previously described^6^. RNA integrity was verified using the Agilent Bioanalyzer 2100 (Agilent Technologies). cDNA was synthesized from total RNA using Superscript III (Invitrogen). Each sample was then sequenced with the HiSeq 2500 to generate 2 × 75-bp paired-end reads. All reads were independently aligned with STAR_2.4.0f17 for sequence alignment against the human genome build hg19, downloaded via the UCSCgenomebrowser (http://hgdownload.soe.ucsc.edu/goldenPath/hg19/bigZips/), and SAMTOOLS v0.1.19 8 for sorting and indexing reads. Cufflinks (2.0.2)9 was used to get the expression values (FPKMS), and Gencode v19 10 GTF file for annotation. Since the sequenced samples from the published data were processed using different library preps, batch normalization was done using ComBat11 from sva bioconductor package 12. The gene counts from htseq-count13 and DESeq2 Bioconductor package14 were used to identify differentially expressed genes. The hypergeometric test and Gene Set Enrichment Analysis (GSEA)v15 was used to identify enriched signatures using the different pathways collection in the MSigDB database 16. We used GSEA pre-ranked method from GSEA for our purpose. Principal Component Analysis (PCA) was performed using the prcomp function of R “stats” package (https://cran.r-project.org/), and visualization was done using ggbiplot package (https://github.com/vqv/ggbiplot). A Wald test was applied for mRNA differential analysis, followed by Benjamini−Hochberg correction for multiple hypothesis testing.

### AR signaling and integrated CRPC-NE score

For each sample, AR signaling was assessed based on the expression levels of 30 genes^6^. The Integrated Neuroendocrine Prostate Cancer (CRPC-NE) score estimates the likelihood of a test sample to be CRPC-NE and it is computed based on a set of 70 genes^6^. The gene set stems from the integration of differentially deleted/amplified and/or expressed and/or methylated genes in CRPC-NE vs CRPC.

### Methylation profiling

Sample preparation, alignment, and enhanced reduced representation bisulfite sequencing (eRRBS) were performed at the WCM Epigenomics Core Facility^50^ Samples profiled by eRRBS included 19 CRPC-Adeno, 15 CRPC-NE, 2 Organoids, and 1 PDOX samples. Only sites covered by at least ten reads were considered for downstream analysis. For each sample, the percentage of methylation per site (beta value) was computed. Ward’s hierarchical clustering of samples was performed by “1-Pearson’s correlation coefficient” as distance measure on the 5% CpG sites showing the highest standard deviation across the cohort.

### Cell line infection and drug treatments

NCI-H660 used in this study were purchased from ATCC and maintained according to the manufacturers’ protocols. Cell authentication was performed using STR analysis and cells were routinely tested for *Mycoplasma* contamination and resulted negative. shEZH2 used in these studies was kindly provided by Dr. Beguelin and Dr. Melnick (WCM) with the following sequence: TATGATGGTTAACGGTGA. shEZH2 and sh scramble were used to infect CRPC-NE organoids. In brief, organoid cells were collected and resuspended with infection media containing Y27632 (Selleck Chemical) and Polybrene (Millipore). Organoids cells were then placed in 24 well-plates and centrifugated at 600*g* at 32 °C for 60min. After centrifugation organoids were incubated at 37 °C overnight and the following day seeded in Matrigel droplets^51^. We used pLKO.1-puro vector and infected cells were selected by puromycin treatment (1 μg/ml).

Cell viability assays were performed on 4000 organoid cells treated with increasing doses of GSK343 (Sigma-Aldrich SML0766) and GSK503 (GSK provider) at the indicated concentrations for 6 or 11 days and Neratinib, Alisertib, Afuresertib, Cobimetinib were purchased from SelleckChem and used in cell viability assays for 6 days. Viability was measured with cell viability assay kit according to the manufacturer’s protocol (CellTiter-Glo, Promega). For RNA extraction (Qiagen Kit) and protein lysate, treatments were conducted for 6 days. For viability assays all the data are expressed as mean ± standard error of the mean (SEM). Multiple sample comparisons were calculated using ANOVA (in GraphPad Prism 6). Differences between values were considered statistically significant at a *p* value of less than 0.05.

### High-throughput drug assay

For high-throughput drug screens, cells were dispensed into 384-well tissue-culture-treated plates at ~30% confluence (500–1200 cells) using a BioTek MultiFlo™. After 24 h, using robotic liquid handling, cells were exposed to 126 unique drugs. Drugs were diluted to a 6-point dose curve incorporating a 3 or 4-fold dilution step in the presence and absence of an IC30 concentration of GSK503. After 6 additional days of incubation, cell viability was assessed using CellTiter-Glo (Promega) and a BioTek Synergy H4 plate reader. All screening plates were subjected to stringent quality control measures, including the *Z* factor calculation. Raw luminescence units (RLU) were then normalized on a per plate basis to the median values of the negative control: DMSO or PBS, depending on the drug solvent. Dose–response curves were then fit to a 4-parameter logistic model using the R “nplr” package version: 0.1–7. Area Under the Curve (AUC), IC50, and Goodness of Fit (GOF) were calculated for each drug.

AUC values were then compared with the SEngine Precision Medicine internal database of a total of 47 primary tumor samples across multiple tumor types, generating an AUC Z-score that we integrated for the prioritization of future drug investigation. The tumor types included prostate, ovarian, breast, gliomatosis cerebri, myxofibrosarcoma, head and neck, thyroid, liver, CML, endometrial, glioblastoma, colorectal, lung, cholangiosarcoma, uterine carcinosarcoma, and neuroblastoma. This method of statistical analysis allows for the detection of unique sensitivities across multiple samples. For the drug combinations study, the top drug combinations were selected through multiple criteria: AUC fold change, AUC differential, AUC combination Z-score, drug target, novelty, and clinical status of drugs.

### Data availability

The RNA-seq and ERRBS data generated during the current study are available through Gene Expression Omnibus (GEO) accession number: GSE112830 with the following sub-series: https://www.ncbi.nlm.nih.gov/geo/query/acc.cgi?acc=GSE112786, https://www.ncbi.nlm.nih.gov/geo/query/acc.cgi?acc=GSE112829. The whole exome sequencing data related to this study are available through Sequence Read Archive (SRA) with accession number SRP138000. The published human data are available through dbGap:phs000909.v.p1 (http://www.cbioportal.org/study?id=nepc_wcm_2016)^6^.

## Electronic supplementary material

Supplementary Information

Peer Review File

Description of Additional Supplementary Files

Supplementary Data 1
