# Supplementary material for: Patient derived organoids to model rare prostate cancer phenotypes
Source: Nat Commun. 2018 Jun 19;9:2404. doi: 10.1038/s41467-018-04495-z (PMC6008438; doi:10.1038/s41467-018-04495-z)
Supplement: Supplementary file 3 — Description of Additional Supplementary Files [file 41467_2018_4495_MOESM3_ESM.pdf]

Description of Additional Supplementary Files

File Name: Supplementary Data 1.

Description: GSEA analysis, *related to Figure 3.* Significant GSEA signatures in organoids comparing shEZH2 versus shscramble and GSK503 treatment versus vehicle with corresponding p-values and FDR.
