# Supplementary material for: Patient derived organoids to model rare prostate cancer phenotypes
Source: Nat Commun. 2018 Jun 19;9:2404. doi: 10.1038/s41467-018-04495-z (PMC6008438; doi:10.1038/s41467-018-04495-z)
Supplement: Supplementary file 1 — Supplementary Information [file 41467_2018_4495_MOESM1_ESM.pdf]

Supplementary Information

Patient Derived Organoids to Model Rare Prostate Cancer Phenotypes

Puca et al.

Supplementary Figures

OWCM155

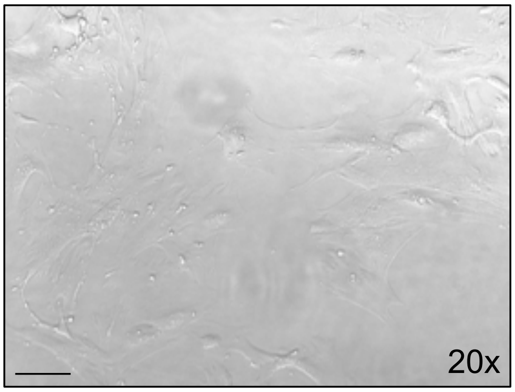

Supplementary Figure 1. Isolation of cancer-associated fibroblasts (CAFs) from OWCM155, a CRPC-NE organoid model, *related to Figure 1.* Bright field image analysis (20x magnification, scale bar 100μm) of cancer associated fibroblasts isolated from 3D organoids culture growing in monolayer.

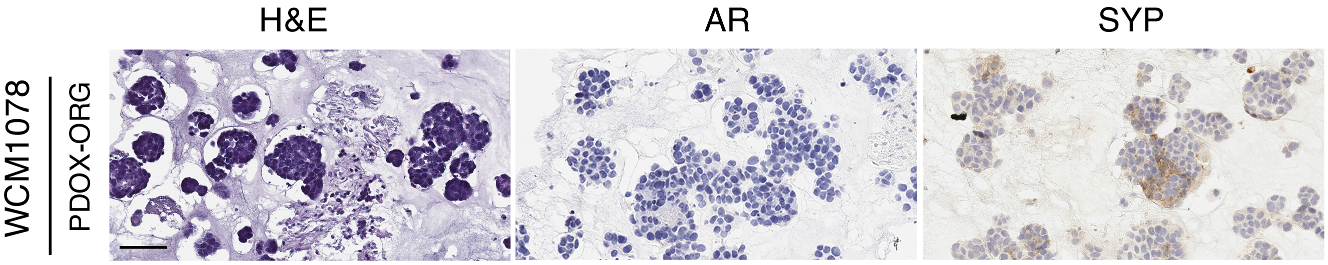

Supplementary Figure 2. PDOX-derived organoids, *related to Figure 1***.** Histology of 3D organoid cultures PDOX-ORG1078 (20X magnification, scare bar 100µm). Samples are stained by IHC with H&E, SYP and AR antibodies.

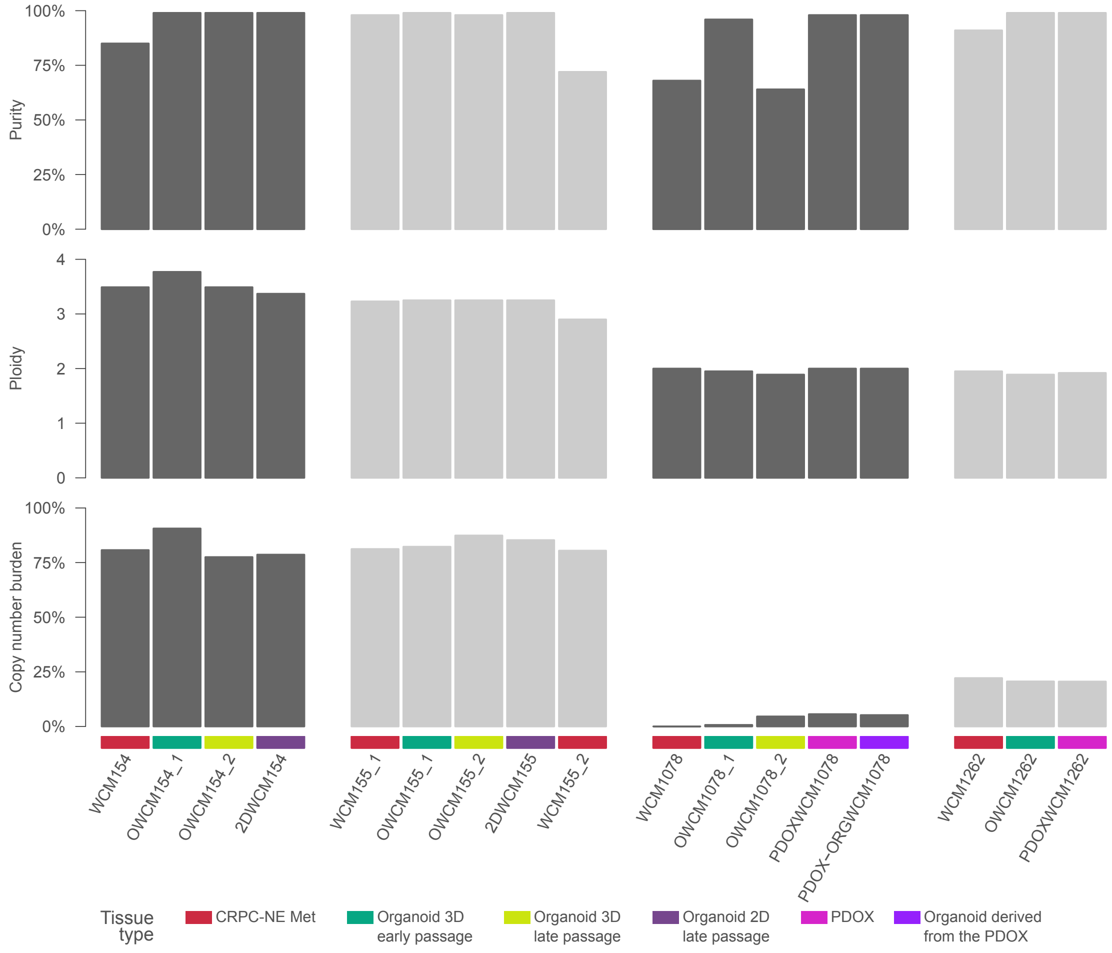

Supplementary Figure 3. Tumor purity, ploidy and copy number burden in models and corresponding patient samples, *related to Figure 2***.** Genomic data from patient-derived models and corresponding patients analyzed using an algorithm called CLONET[1] show similar levels of purity (median 98%), ploidy and copy number burden.

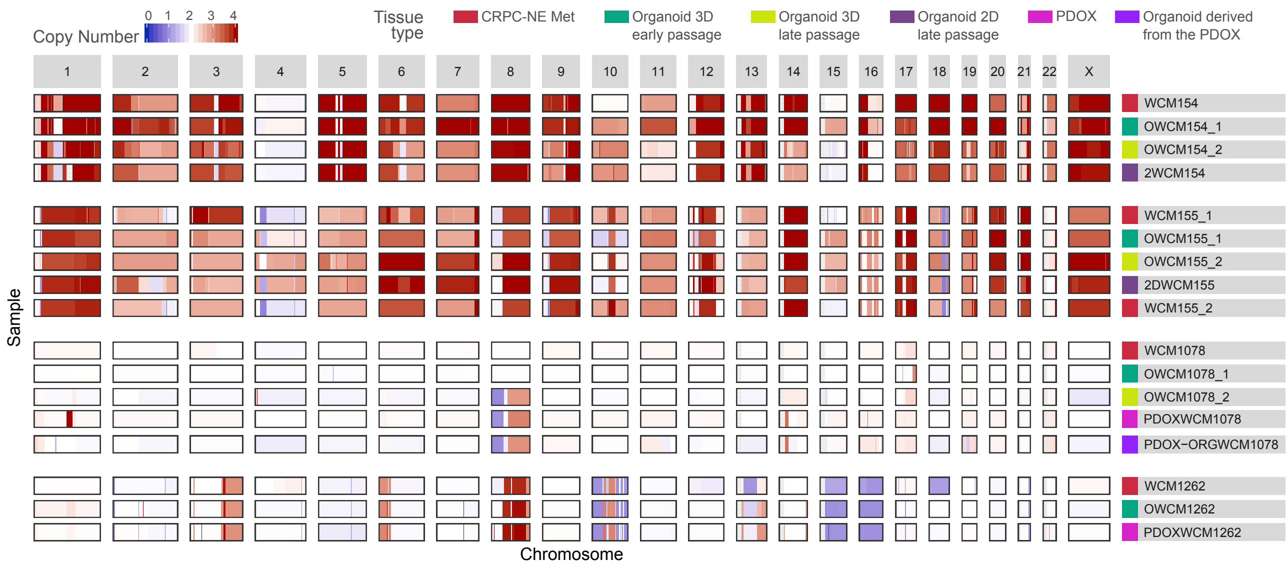

Supplementary Figure 4. Copy number profiles, *related to Figure 2.* Copy number profiles corrected by ploidy and purity across matched tissue, organoids, and corresponding xenografts. WCM154 and WCM155 demonstrate whole genome duplication which is retained across sample types, while WCM1078 and WCM1262 do not. Genomic alterations were conserved along the whole exome between tissue and organoids and PDOXs in all 4 cases. In case WCM1078, there was one area of the genome (at chromosome 8; a region typically altered in prostate cancer) that may have been selected for in late passage organoids and PDOX models possibly due to sub-clonal selection.

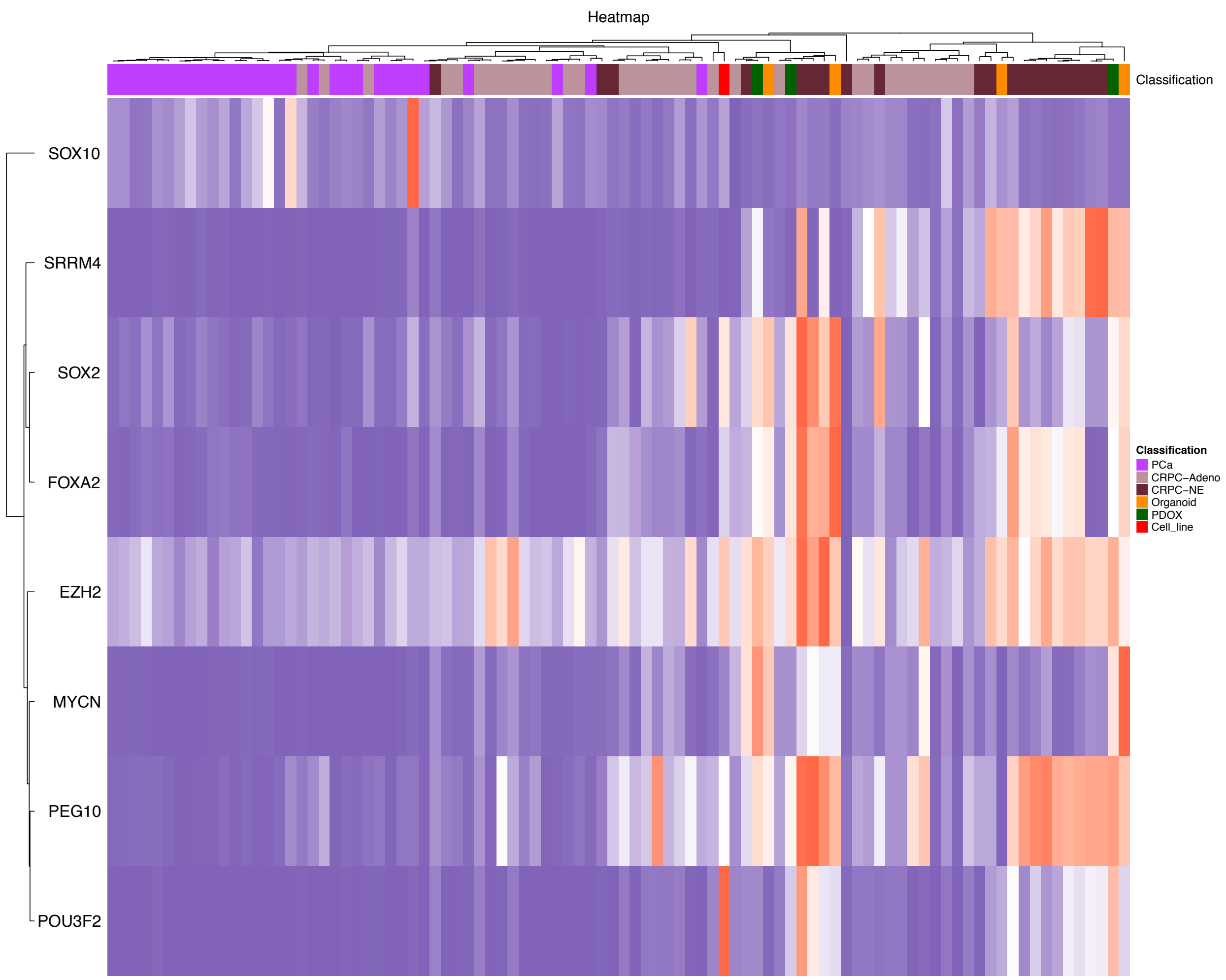

Supplementary Figure 5. Cluster analysis of CRPC-NE associated genes *related to Figure 2.* Unsupervised analysis of published genes involved in CRPC-NE using a cohort of PCA (pink), CPRC-Adeno (mauve) and CRPC-NE (brown) patients (Beltran et el., 2016)[2] and the CRPC-NE patient derived organoids (orange), PDOX (green) and NCI-H660 cell line (red), as control.

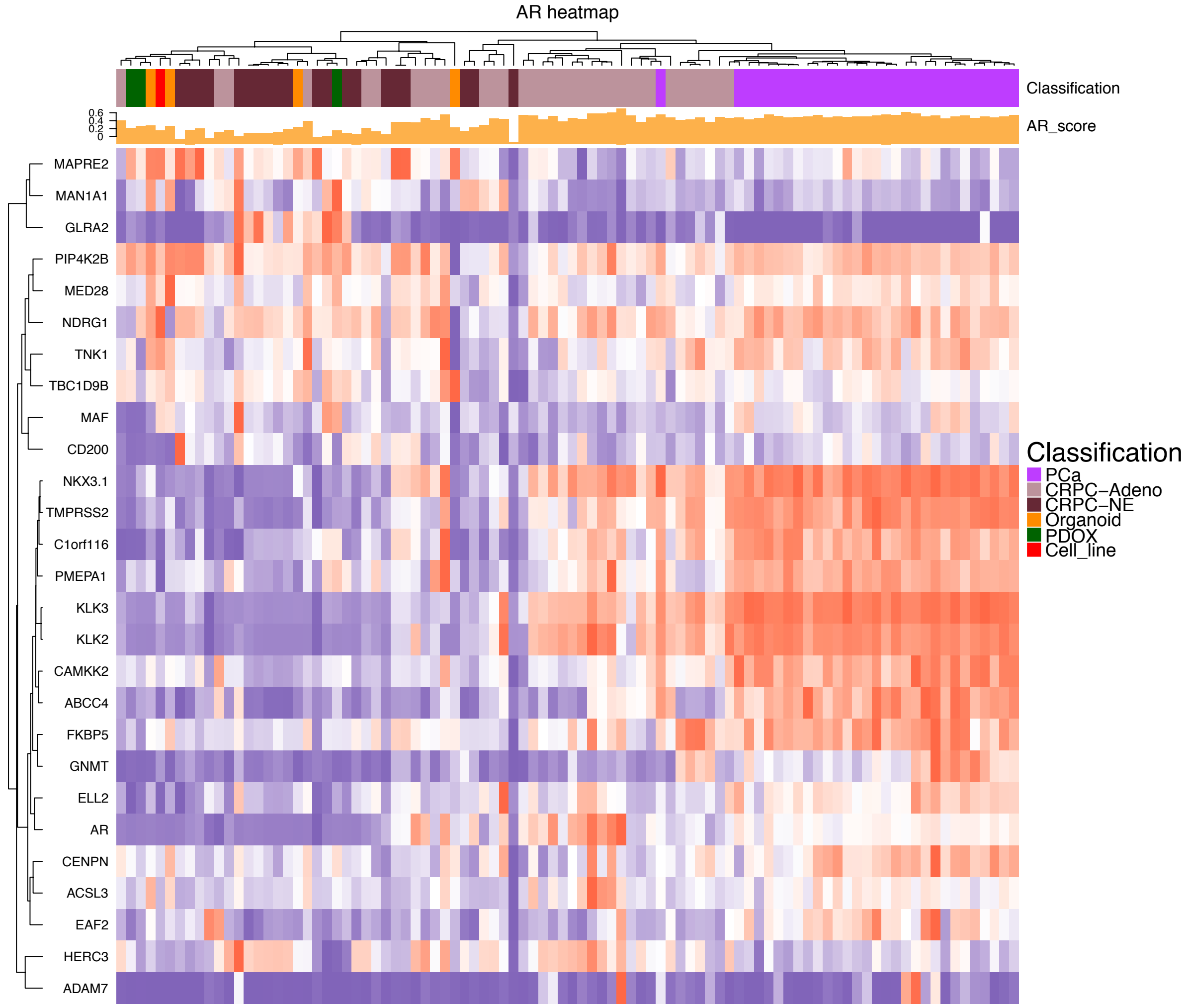

Supplementary Figure 6. Cluster analysis of AR signaling genes, *related to Figure 2.* Unsupervised analysis of genes involved in AR signaling (30 genes) using a cohort of PCA (pink), CPRC-Adeno (mauve) and CRPC-NE (brown) patients (Beltran et el., 2016)[2] and the CRPC-NE patient derived organoids (orange), PDOX (green) and NCI-H660 cell line (red), as control. The top yellow barplot represents the AR score calculated according to Beltran et al., 2016[2].

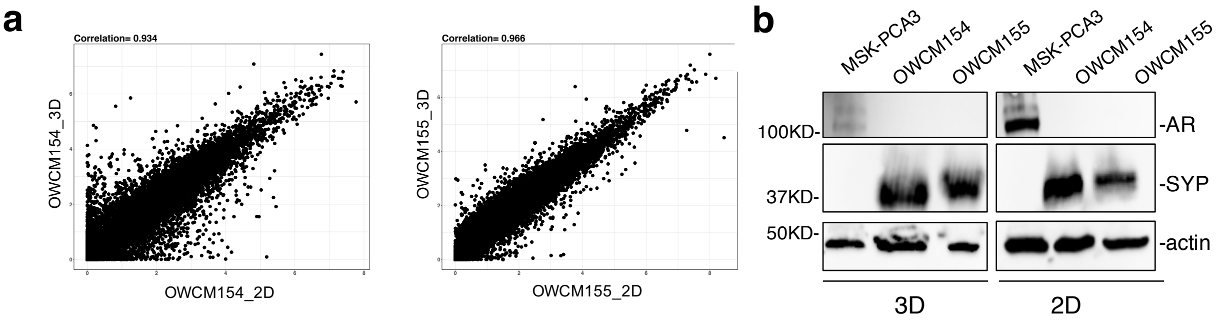

Supplementary Figure 7. Correlation of 3D and 2D models by transcriptomic analysis and protein expression, *related to Figure 2.* **a.** Transcriptomic analysis of organoids showed no significant differences in gene expression between 2D and 3D cultures (correlation coefficient 0.934 for OWCM154 and 0.966 for OWCM155). **b.** Western blot analysis of 3D organoids and the corresponding 2D version shows that both types of models express high level of synaptophysin and lack of AR expression. Actin is used as loading control.

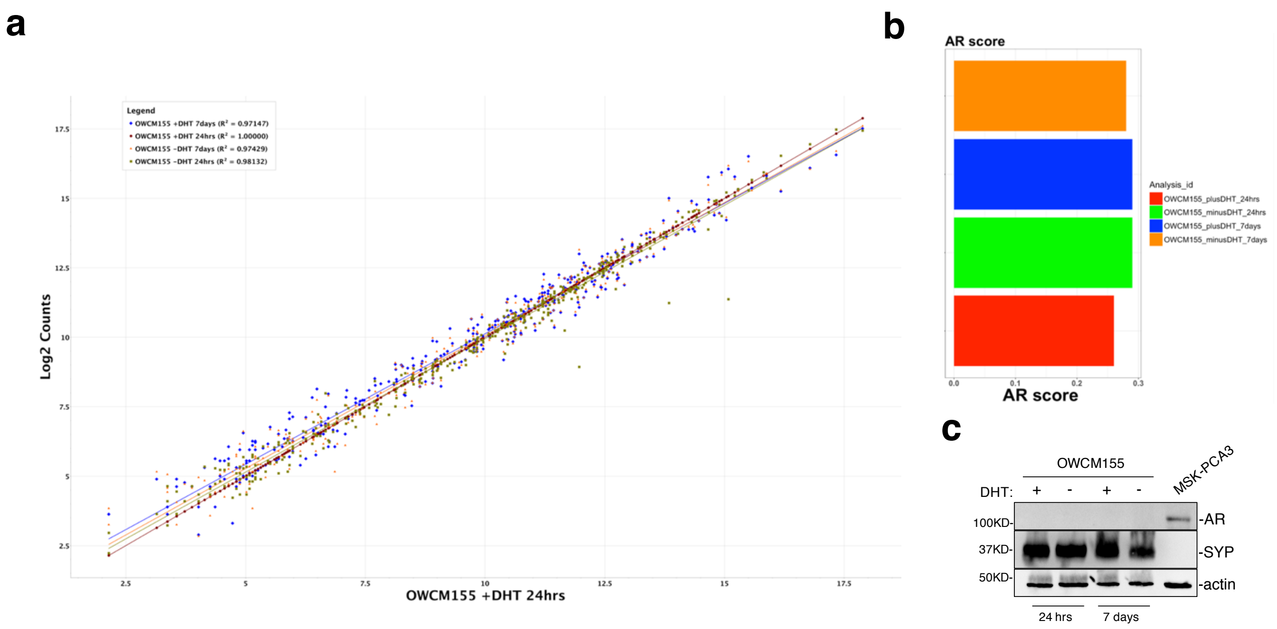

Supplementary Figure 8. Effect of organoid media without DHT on transcriptomic profile, AR score and AR expression, *related to Figure 2.* **a.** Nanostring mRNA analysis of OWCM155 DHT 24 hrs, OWCM155 -DHT 24 hrs, OWCM155 DHT 7days, OWCM155 -DHT 7days. Correlation of nanostring mRNA counts in +/- DHT media conditions was evaluated (r=0.97). **b.** AR signaling score evaluated in +/- DHT media conditions shows no significant differences. **c.** Western blot analysis shows that CRPC-NE organoids, OWCM155, lacked AR expression with high SYP in all the conditions evaluated. MSK-PCA3 is used as control of AR expression.

a

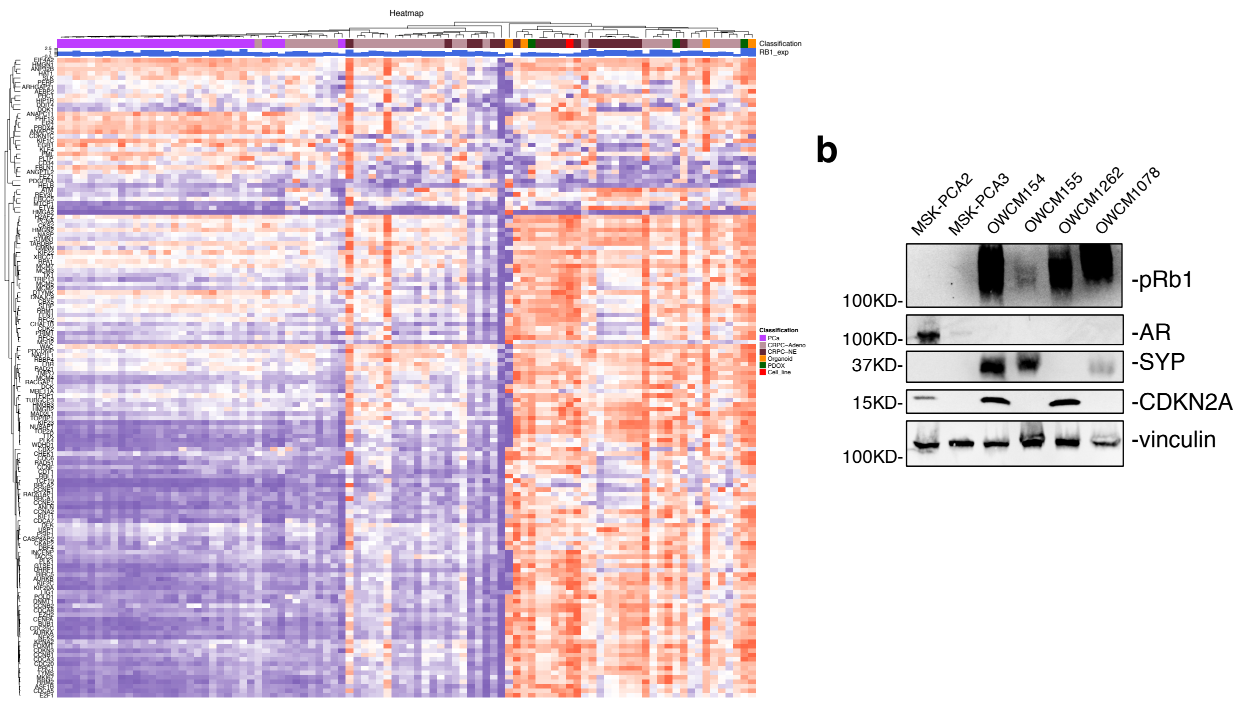

Supplementary Figure 9. Dysregulation of RB signaling pathway, *related to Figure 2.* **a.** Cluster analysis of genes associated to RB functional loss from Ertel et al.[3] using a cohort of PCA (pink), CPRC-Adeno (mauve) and CRPC-NE (brown) patients (Beltran et el., 2016)[2] and the CRPC-NE patient derived organoids (orange), PDOX (green) and NCI-H660 cell line (red) as control with relative RB1 mRNA expression depicted in blue. **b.** Western blot of phospho-RB1 and CDKN2A, relevant proteins associated to RB1 pathway disruption. SYP and AR antibodies were used to confirm CRPC-NE features of the developed organoids. MSK-PCA2 and MSK-PCA3 (CRPC-Adeno organoids) are used as controls.

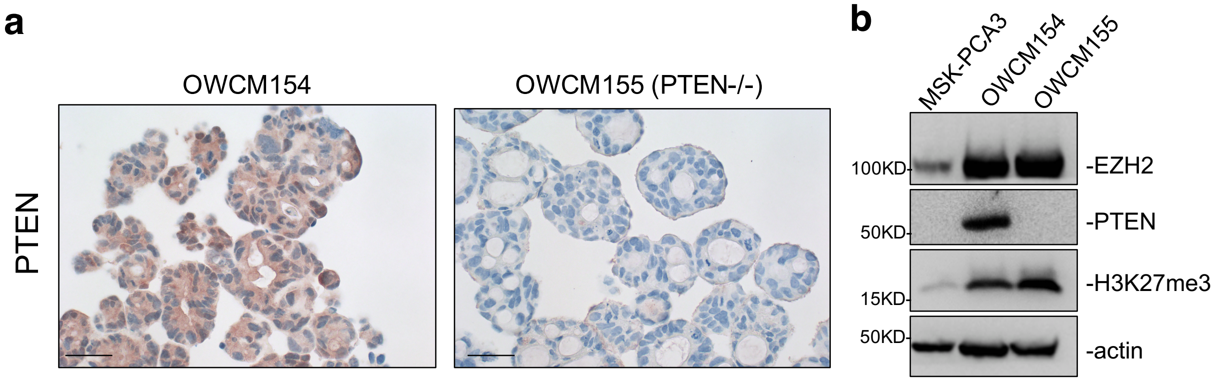

Supplementary Figure 10. Validation of PTEN genomic loss by IHC and Western Blot in OWCM155, *related to Figure 2*. **a.** Histology of 3D organoid cultures OWCM155 and OWCM154 stained with PTEN antibody to validate PTEN genomic loss (40X magnification, scare bar 50μm). **b.** Western blot analysis highlighting features of OWCM154 and OWCM155 compared to CRPR-Adeno organoids (MSK-PCA3) using EZH2, H2K27me3 and PTEN antibodies. Actin is used as loading control.

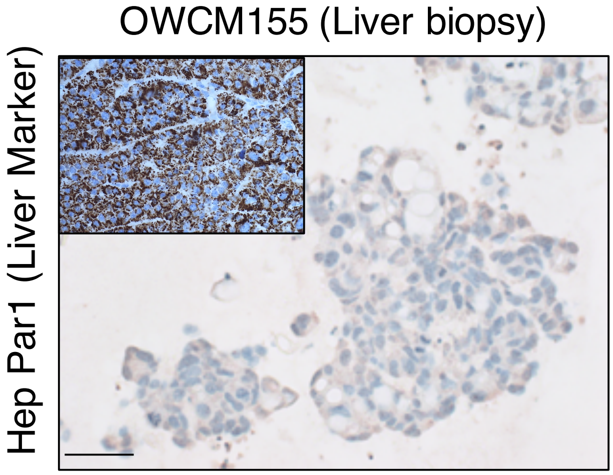

Supplementary Figure 11. Tumor specificity in liver biopsy organoids evaluated by Hep Par 1 staining *related to Figure 2***.** 3D organoid culture OWCM155 (40x, scale bar 50µm) derived from liver biopsy was stained with Hep Par1 antibody, an established liver marker. Benign liver tissue is used as positive control (inset, 400x) showing strong and diffuse granular cytoplasmic staining. The image shows absence of benign liver cells contamination in tumor organoid culture.

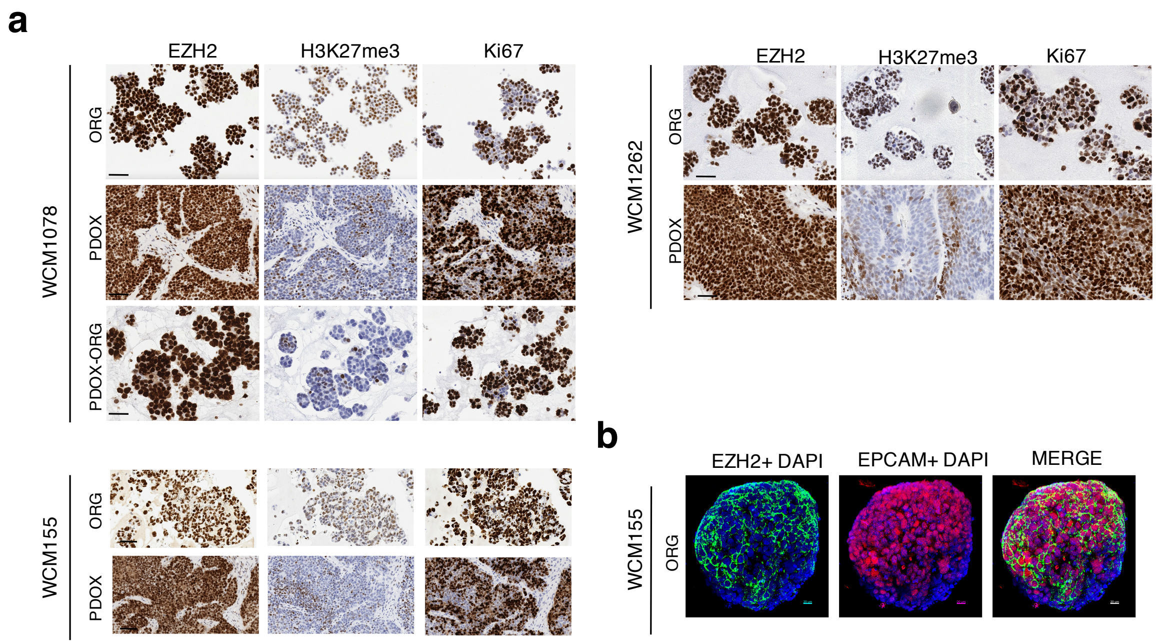

Supplementary Figure 12. EZH2, H3K27me3 and Ki67 expression in additional CRPC-NE models, *related to Figure 3*. **a.** Histology of 3D organoid cultures and corresponding PDOXs, (20X magnification, scare bar 100μm) stained with EZH2, H3K27me3 and KI67 antibodies. **b.** Immunofluorescence staining of OWCM155 using EZH2 (Alexa-555) and APC anti-human EPCAM and 40,6-diamidino-2-phenylindole, dihydrochloride (DAPI). Z-stacks are obtained using a Zeiss confocal microscope (LSM510; Carl Zeiss Microscopy). 3D Images are obtained combining the Z-stacks using Imaris software (scale bar 20μm).

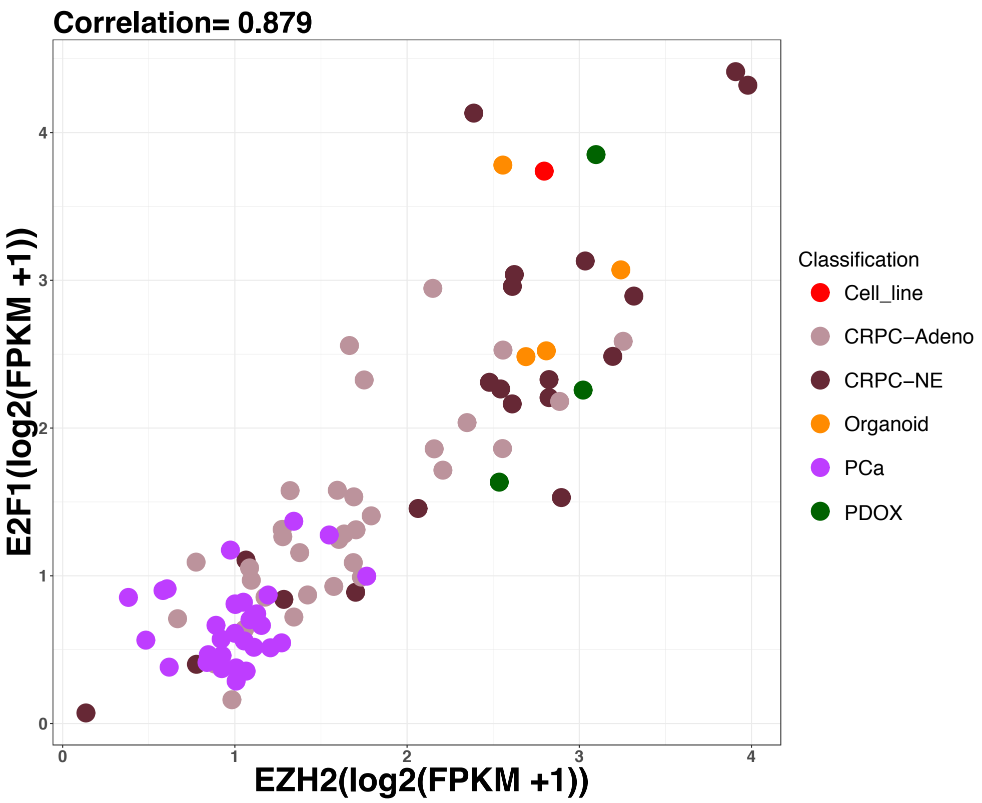

Supplementary Figure 13. Correlation of EZH2 mRNA expression and E2F1 in prostate cancer patient cohort and models, *related to Figure 3.* Transcriptomic correlation between EZH2 and E2F1 using a cohort of PCA (pink), CPRC-Adeno (mauve) and CRPC-NE (brown) patients (Beltran et el., 2016)[2] and the CRPC-NE patient derived organoids (orange), PDOX (green) and NCI-H660 cell line (red) as control reveals that the expression of EZH2 and E2F1 highly correlate in CRPC-NE patients and models (r=0.879).

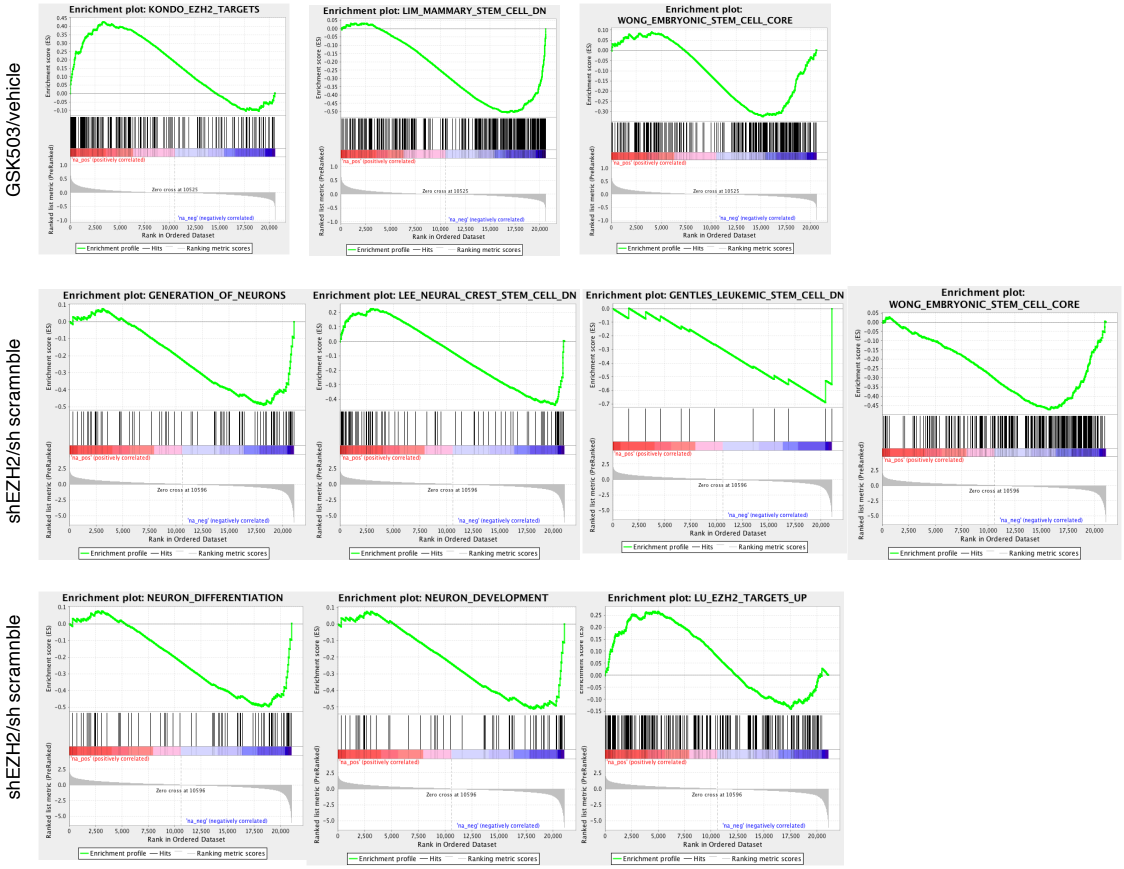

Supplementary Figure 14. GSEA plots of significant Stem Cell, Neuronal & EZH2 signatures identified in shEZH2 versus shscramble and EZH2 inhibitor versus vehicle, *related to Figure 3.*

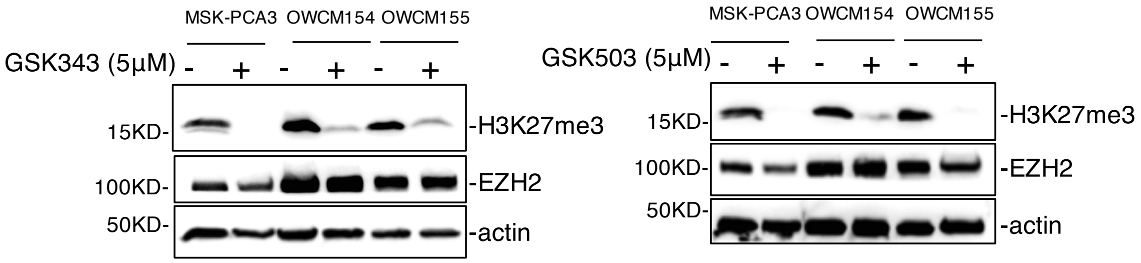

Supplementary Figure 15. Effects of GSK343 and GSK503 on H3K27me3, *related to Figure 3.* Western blot analysis of MSK-PCA3, OWCM154 and OWCM155 was performed. EZH2, H3K27me3 antibodies were used. Actin was used as loading control. H3K27me3 expression is reduced when organoids are treated with either GSK343 or GSK503.

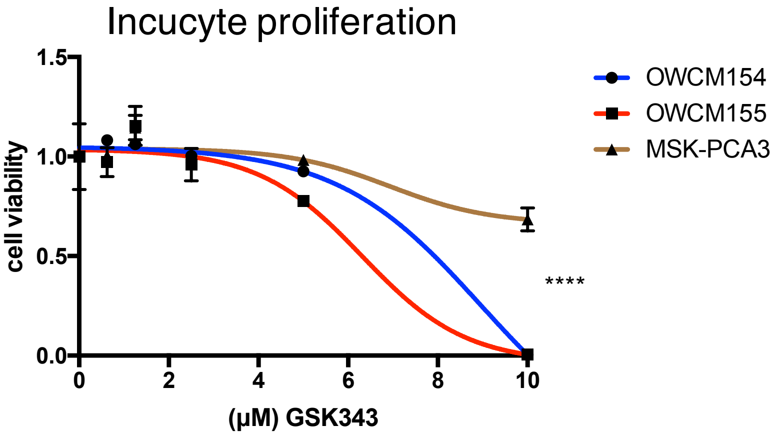

Supplementary Figure 16. EZH2 inhibitor effects on proliferation of OWCM154 and OWCM155 compared to CRPC-Adeno, MSK-PCA3, evaluated through the Incucyte system, *related to Figure 3***.** Proliferation was measured as confluency of cells from day 0 to day 11 during the treatment with GSK343 (n = 2, for each dose. Error bars, s.e.m). Values generated by the Incucyte system have been analyzed using Two-way ANOVA test. **** $p < 0.0001$ (MSK-PCA3 vs OWCM154, MSK-PCA3 vs OWCM155).

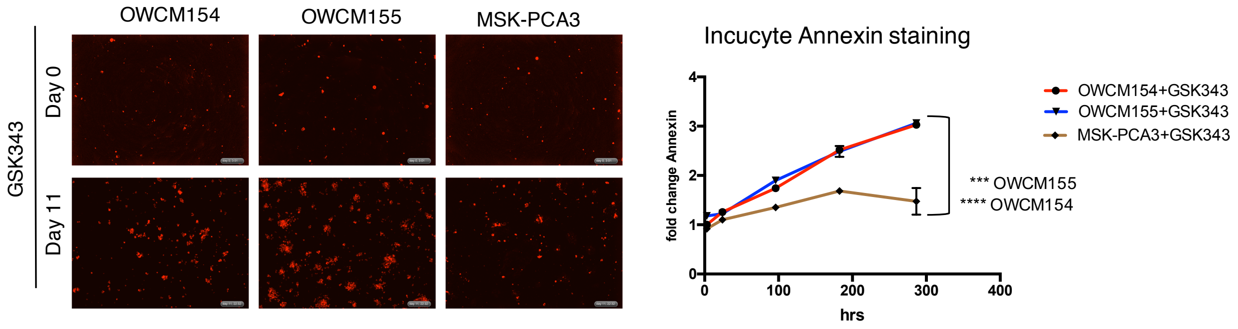

Supplementary Figure 17. EZH2 inhibitor effects on apoptosis of OWCM154 and OWCM155 compared to CRPC-Adeno, MSK-PCA3, evaluated through the Annexin staining with Incucyte system, *related to Figure 3.* Annexin-RFP staining representative images and Annexin fold change graph in OWCM154, OWCM155, MSK-PCA3 evaluated for 11 days after treatment with vehicle or GSK343 (10uM) measured with the Incucyte system (n=2, for each time point. Error bars, s.e.m). Two-way ANOVA test is used. ****p<0.0001 for OWCM154 and ***p<0.001 for OWCM155.

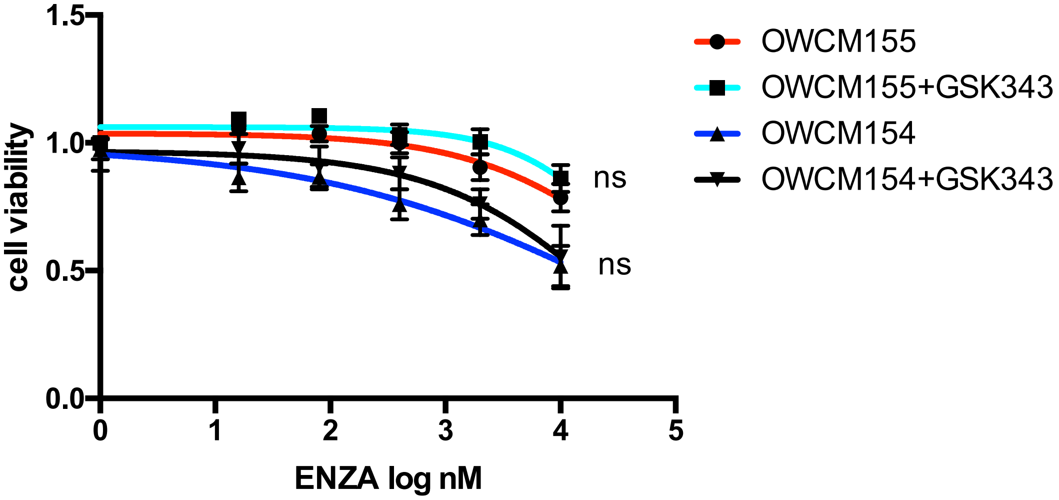

Supplementary Figure 18. Cell viability in CRPC-NE organoids co-treated with EZH2 inhibitor and enzalutamide, *related to Figure 3***.** Cell viability assay performed on OWCM154 and OWCM155 organoids treated with increasing doses of enzalutamide (ENZA) and a fixed dose of GSK343 (5µM) for 6 days (n=9, for dose. Error bars, s.e.m.). Two-way ANOVA test is used (ns= not significant).

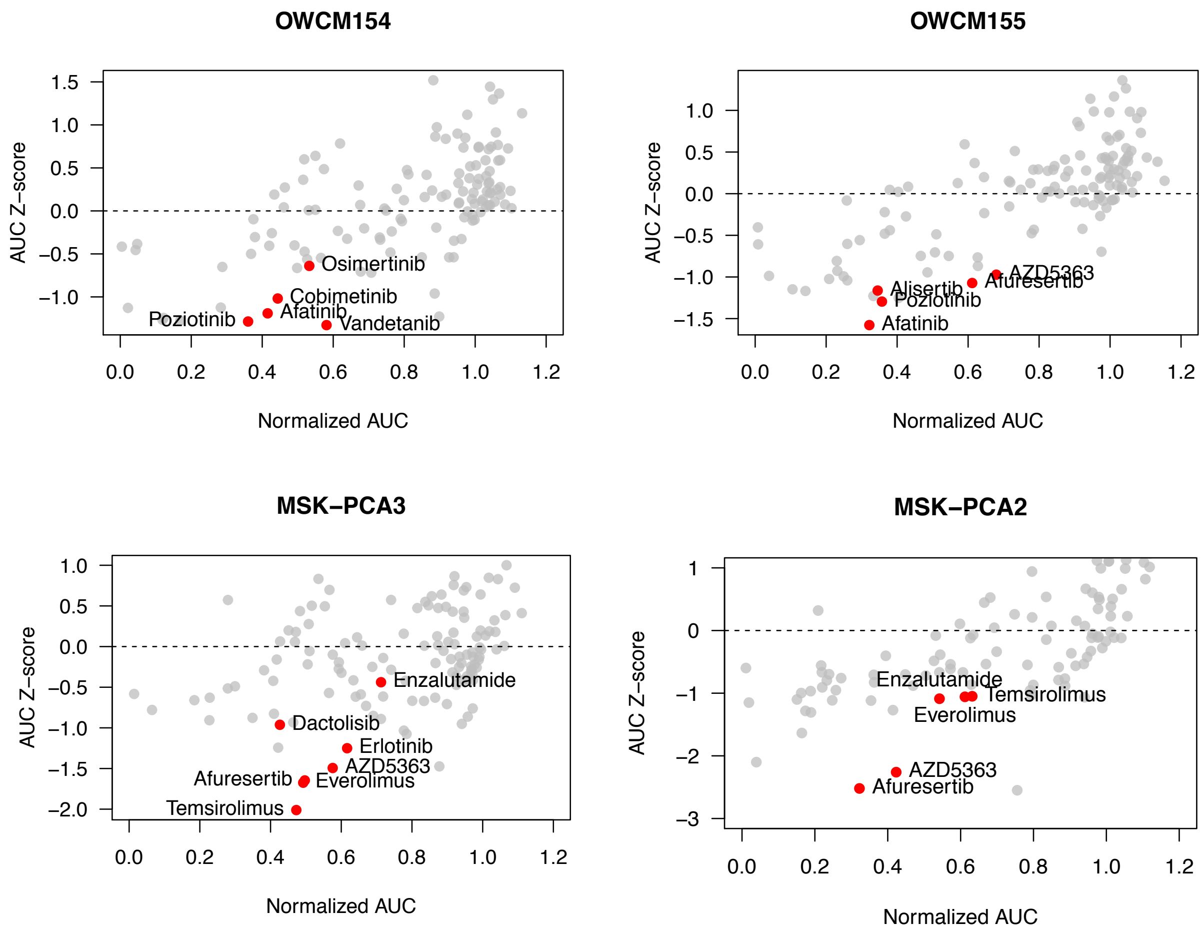

Supplementary Figure 19. Individual organoids response to single agent drug screening, *related to Figure 4***.** High-throughput drug screening analysis based on Area under the curve and Z-score (described in method) for each organoid tested. The top scoring single agents for each organoid are highlighted in red.

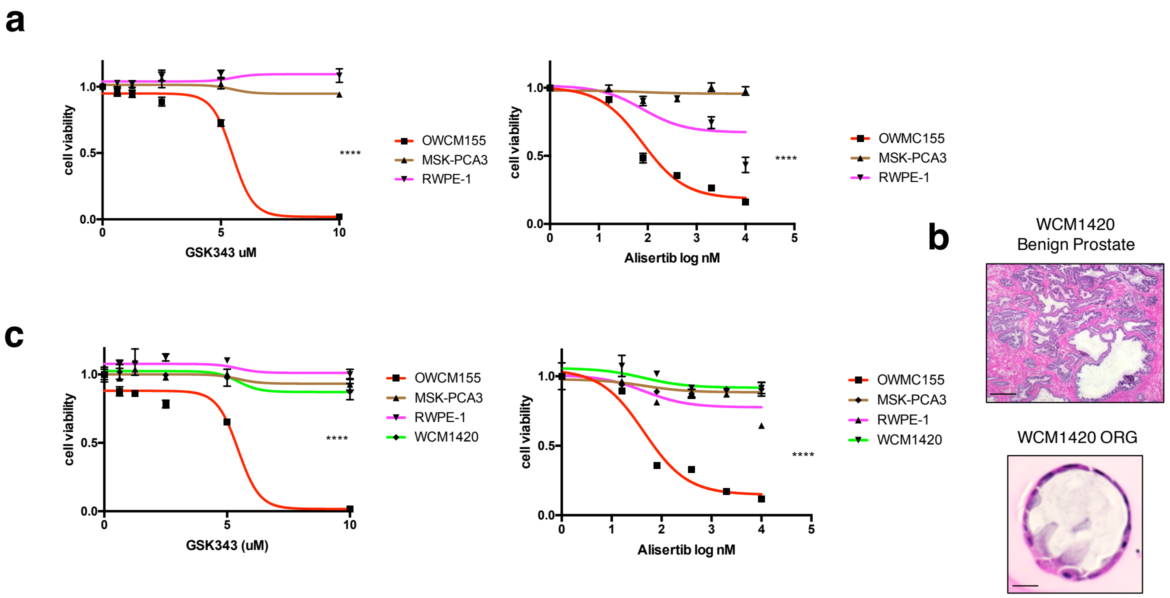

Supplementary Figure 20: Effects of Alisertib and GSK343 on RWPE-1, a benign prostate cell line, and benign prostate organoids, *related to Figure 4.* **a.** Cell viability assay performed on OWCM154, OWCM155 and MSK-PCA3 organoids and RWPE-1 benign prostate cell line treated with increasing doses of GSK343 or Alisertib for 6 days) (n=9, for each dose. Error bars, s.e.m.). Two-way ANOVA test is used **** $p < 0.0001$ (RWPE-1 vs OWCM155 for GSK343 and Alisertib treatment) **b.** H&E staining of a benign prostate tissue (WCM1420) and corresponding organoids (40x, scale bar 50µm). **c.** Cell viability assay performed on OWCM154, OWCM155 and MSK-PCA3 organoids, RWPE-1 and WCM1420 ORG (n=3, for each dose. Error bars, s.e.m) treated with increasing doses of GSK343 or Alisertib for 6 days (Two-way ANOVA test is used **** $p < 0.0001$ (WCM1420 vs OWCM155 for GSK343 and Alisertib treatment).

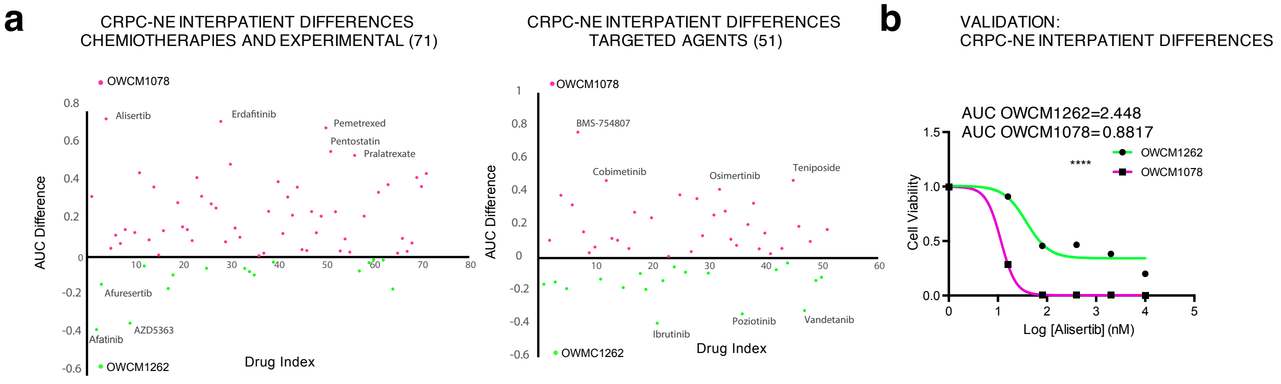

Supplementary Figure 21. 3D-single agent drug screen and validation for additional CRPC-NE organoids, *related to Figure 4.* **a.** OWCM1078 and OWCM1262 screening results are plotted based of the Area under the curve (AUC) difference in drug responses. **b.** Cell viability assay performed on OWCM1078 and OWCM1262 organoids treated with increasing doses of Alisertib for 6 days (n=9, for each dose. Error bars, s.e.m.). AUC difference is calculated and Two-way ANOVA test is used: p<0.0001 (****).

a

b

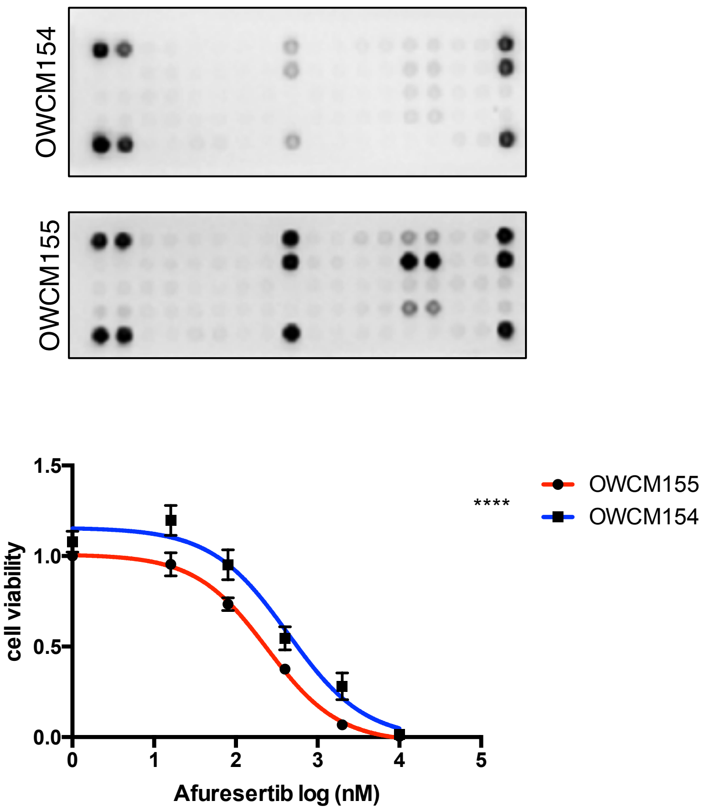

Supplementary Figure 22. PTEN genomic loss and response to AKT inhibitor (Afuresertib) in OWCM155, *related to Figure 4.* **a.** Receptor tyrosine kinase array was performed using proteins from OWCM155 and OWCM154. Positive and negative controls are on each side of the array. Strong signals for Phospho-AKT and phospho-S6 signals (black spots) are observed in OWCM155 compared to OWCM154. **b.** Cell viability assay using OWCM154 and OWCM155 with increasing doses of AKT inhibitor (Afuresertib) for 6 days (n=9, for each dose. Error bars, s.e.m.). Two-way ANOVA test is used. $p< 0.0001$ (****)

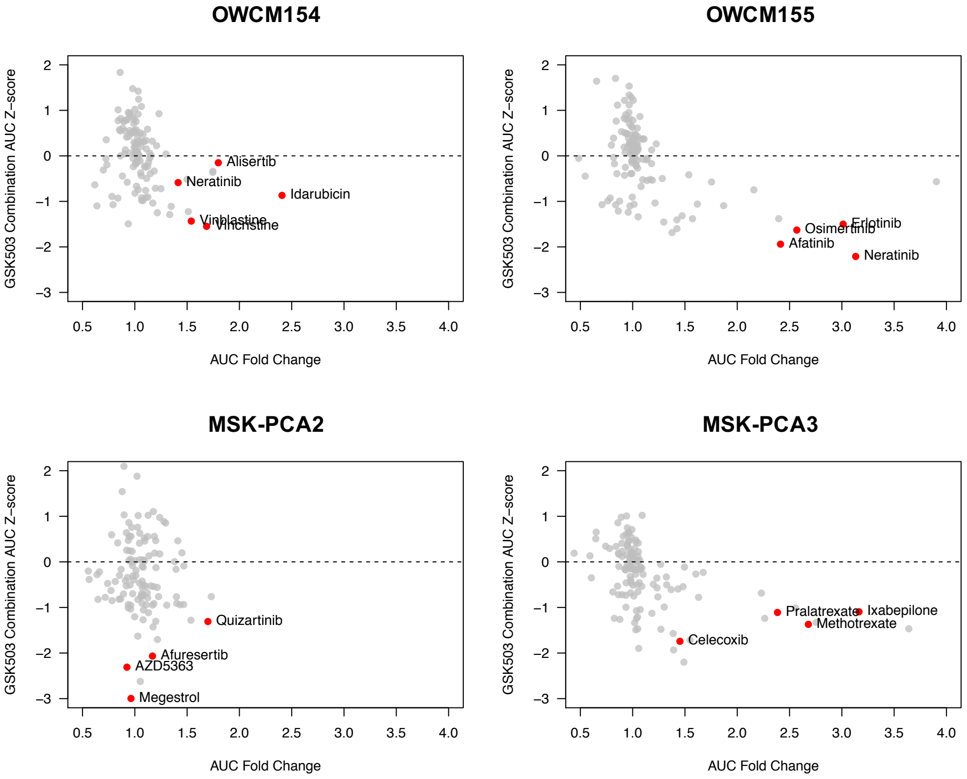

Supplementary Figure 23. Individual organoid responses to the drug screening in combination with EZH2 inhibitor, *related to Figure 4.* High-throughput drug screening analysis based on Area under the curve and Z-score (described in method) for each organoid tested in combination with GSK503 IC30. The top scoring single agents for each organoid are highlighted in red.

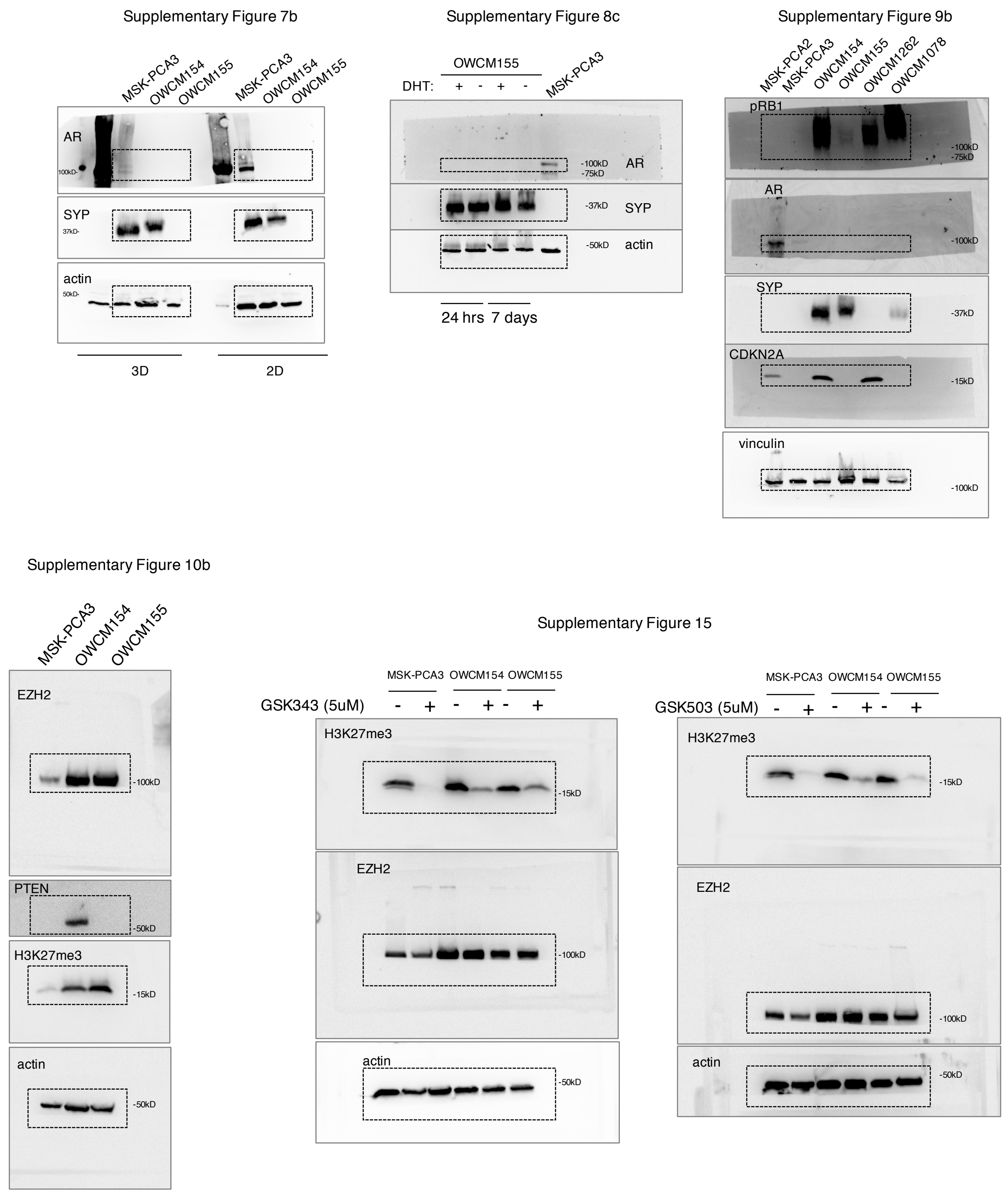

Supplementary Figure 24. Full blots

Supplementary Methods

Incucyte proliferation and Annexin

The IncuCyte ZOOM system (Essen BioScience) was used to image the effects of GS343 on cellular proliferation and apoptosis. OWCM154, OWCM155, MSK-PCA3 prostate organoid cells were plated in duplicate in a 96-well plate in 100 uL of media and increasing doses of GSK343 were applied. After 30 minutes at room temperature for even distribution, the cells were placed in the IncuCyte and incubated at 37 C and 5% CO2 overnight, with phase contrast images taken every 4 hours at 10x objective. At 24 hours post initial plating, the plate was removed from the incubator and GSK343 and Incucyte Annexin V Reagent (Essen BioScience) working solution was prepared at a 1:67 dilution in organoids media, containing 3x final assay drug treatment concentrations. 50 uL of the working Annexin V and drug treatment solution was added to each well of the plate for a total of 150 uL of solution per well. Apoptotic and confluence signal measurement was performed every 4 hours for approximately 260 hours. Data were analyzed using GraphPad Prism 6.

High Throughput 3D drug screen method

For high throughput 3D drug screens, 384-well plates (Perkin Elmer 6007680) were coated with a solution of 50% BD Matrigel and 50% tissue specific growth media with Felix robot (Analytik Jena). 500-1,200 cells depending on sample were added to achieve ~30% confluence (BioTek MultiFlo™ FX). Assay plates were covered with a gas permeable membrane (ThermoScientific AB0718) to prevent media evaporation and incubated for 72hrs to promote organoid formation. 126 unique drugs were added at a 6-point 3-fold dilution in arrayed format (Single Agent Screens) and in the presence or absence of a sensitizing agent at IC30 (Combination Screens). Organoids were exposed to these drugs for an additional 96 hours. Cell viability was assessed using CellTiter-Glo (Promega) and a BioTek Synergy H4 plate reader.

Raw luminescence units (RLU) were normalized on a per plate basis to the median values of the vehicle control. Dose response curves were fit to a 4-parameter logistic model (R ‘nplr’ package version: 0.1-7). Area Under the Curve (AUC), IC50, and Goodness of Fit (GOF) were calculated for each

drug response. AUC values were then compared with the SEngine Precision Medicine internal database of a total of 55 primary tumor samples across multiple tumor types allowing for the detection of unique sensitivities across multiple samples. Downstream analysis (Combination Screens) may include AUC fold change, AUC differential, AUC combination Z-score, drug target, novelty, and clinical status of drugs. Top drug combinations were selected on these criteria.

RTK Signaling Antibody Array

RTK Array (Cell Signaling, 7982) was used for detection of 28 receptor tyrosine kinases and 11 signaling nodes with phosphorylated residues. The assay has been performed according to the manufacturer's protocol. Briefly, cell lysed is incubated on the slides followed with biotinylated detection antibody cocktail. Streptavidin-conjugated HRP reagent is used to visualized the spot signals by chemiluminescence. Images have been captured on a digital imaging system after the addition of the Lumi/GLO/Peroxide Reagent.

RB heatmap

RB heatmap was created using RB loss signature [3].

Nanostring

A custom designed nanostring panel with 361 probes from genes previously associated to CRPR-NE phenotype[2] was developed. mRNA was extracted using Qiagen RNeasy Min plus kit, following manufacturer's instructions and QC was performed on nanodrop. Samples were run on the NanoString nCounter Analysis System according to the manufacturer's directions and previously described in Beltran et al., 2016. Raw count data was normalized using the nSolver analysis software version 3.0 and a correlation plot was generated. Normalization is performed according to positive- and negative-control probes and the geometric mean of the six housekeeping primers.

Supplementary References

1 Prandi, D. *et al.* Unraveling the clonal hierarchy of somatic genomic aberrations. *Genome biology* **15**, 439, doi:10.1186/s13059-014-0439-6 (2014).

2 Beltran, H. *et al.* Divergent clonal evolution of castration-resistant neuroendocrine prostate cancer. *Nature medicine* **22**, 298-305, doi:10.1038/nm.4045 (2016).

3 Ertel, A. *et al.* RB-pathway disruption in breast cancer: differential association with disease subtypes, disease-specific prognosis and therapeutic response. *Cell cycle (Georgetown, Tex.)* **9**, 4153-4163, doi:10.4161/cc.9.20.13454 (2010).
