# Supplementary material for: Patient derived organoids to model rare prostate cancer phenotypes
Source: Nat Commun. 2018 Jun 19;9:2404. doi: 10.1038/s41467-018-04495-z (PMC6008438; doi:10.1038/s41467-018-04495-z)
Supplement: Supplementary file 2 — Peer Review File [file 41467_2018_4495_MOESM2_ESM.pdf]

Reviewers' comments:

Reviewer #1 (Expertise: Prostate Cancer, Remarks to the Author):

By using an in vitro organoid culture and an in vivo PDX model, the current study recapitulated neuroendocrine prostate cancer from patient specimens, thoroughly characterized them as valid models and then utilized these models to evaluate the effects of EZH2 inhibitor in reverting the neuroendocrine tumor back towards an adenocarcinoma phenotype. This high-throughput organoid drug screening is extremely valuable in identifying effective single agents and novel drug combinations to treat this currently undruggable tumor type in advanced PCa. This approach mayl provide greater therapeutic opportunities for castration resistant and progressive neuroendocrine prostate cancers and provide a platform for interrogating the molecular underpinnings of adenocarcinoma transdifferentiation towards neuroendocrine tumors in advanced castration-resistant prostate cancer.
Despite the significant stengths of the study, there are multiple issues that need to be addressed.
1. The opening sentence of the paper is inaccurate; PCa is the most common non-cutaneous cancer and the third leading cause of cancer-related deaths in United States men.
2. Results, first sentence; please make it clear that the 4 organoids were developed from different patients. While stated in the Methods section, it should be clear upon reading the paper without looking that up.
3. For all of the figures, it is very cumbersome to read through the complicated and inconsistent labeling of the models that include WCM in each label. This was particularly difficult for trying to figure out the meaning of the schematic in Fig 1C, where the tissue is referred to as WCM, the organoid as OWCM, the PDX as PDXWCM, etc. Please re-label this clearly as tissue, organoid, PDX and PDX-derived organoid.
4. Further, the organoid-derived PDX really needs to be labeled and referred to as such rather than simply PDX (e.g. PODX or PDOX). The term PDX conventionally refers to a xenograft derived directly from grafting a patient specimen. While the grafts from organoids may recapitulate some (or all) of the original tumor features, it is critical not to confuse the literature.
5. Page 6: How does a cytology smear confirm that the cells are tumor?
6. Similarly, a few sentences later, the authors state that the PDXs retained the histologic and morphologic features of the parental tumors. Which features? The histology images are way too small to confirm or refute these vague statements. As a result, the data are not convincing.
7. The Supplemental figures are presented out-of-order in the paper. Please correct.
8. Page 7: the authors claim that the organoids and PDXs clustered based on their shared expression of gene signatures… However, except for one sample of >20 in Figure 2D, there is no PDX data present.
9. Figure 2C: The heat map is labeled as NECP whereas the term CRPC-NE is used in the text. Consistency is essential with so many models and derivations in this dataset.
10. Page 8: The authors make a concluding statement that the organoids are clinically relevant systems to further study CRPC-NE pathogenesis. What data supports this statement regarding pathogenesis?
11. The EZH2 inhibitor GSK343 treatment resulted in a preferential decrease in the viability of CRPC-NE organoids. EZH2 knockdown by shRNA decreased the expression of classical CRPC-NE markers.

Did EZH2 knockdown by shRNA result in a similar effect on organoids growth as treatment with the EZH2 inhibitor?
12. To increase the clinical relevance of this methodologic paper, the in vivo PDX model should be employed to test the effects of EZH2 inhibitor and other single or combination therapies.
13. Page 24 under Methods: For each PDX graft, “1.5 million” of organoids were injected sounds impossible. Do the authors mean 1.5 million cells from organoid cultures? How long do the PDXs grafts grow under SQ? Are NOD SCID mice males or females? Age? Animal protocol? Housing? These need to be described in more detail.
14. Dr. Vessella’s group has developed many prostate cancer patient-derived xenograft lines including neuroendocrine cancer line LuCaP49 with intensive publications. This work needs to be discussed.

15. There are some typos and grammar issues scattered throughout that the authors need to better edit.

Reviewer #2 (Expertise: Prostate cancer genomics, Remarks to the Author):

In the manuscript by Puca et al, the authors report their work in the development and characterization of patient derived organoids to model small cell prostate cancer. A few such models were successfully established, and the authors demonstrate genomic, transcriptomic and epigenomic concordance between
organoids and their corresponding patient tumors. Finally, the authors utilize these organoids to understand the biologic role of the epigenetic modifier EZH2 in driving molecular programs associated with lineage plasticity and neuroendocrine progression. High-throughput organoid drug screening identified single agents and novel drug combinations suggesting repurposing opportunites.

Although patient-derived organoids have been used in the study of many cancers including prostate cancer, having small cell prostate cancer model is highly valuable. In this regard, this group, which has been leading prostate cancer genomics studies, has made important new contributions to the field. The EZH2 study is less novel. However, it does help to validate the models and gives us an opportunity to look at the potential of using EZH2 inhibitor for the treatment of small cell prostate carcinoma. The high-throughput drug screening represents an important step to introduce the obtained model resources into translational utility.

Therefore, despite some weakness in novelty, I still believe this represents strong work that will lead us a step closer towards better understanding and potential therapeutic strategy of small cell carcinoma. I have a few suggestions for the authors:

1. Terminology: The names used to describe the tumor pathology are somewhat inconsistent. Throughout the manuscript, the authors used "neuroendocrine prostate cancer", " small cell neuroendocrine
carcinoma", "high-grade carcinoma with extensive neuroendocrine differentiation". This is confusing

to readers. Do they all mean the same tumor or do they have different meanings? I understand there is a historic reason for this confusion, and a recent paper (reference 9) failed to adequately address this. However, the authors have a responsibility to use a consistent term if they are talking about the same tumors because to pathologists, there are adenocarcinomas and small cell carcinomas (or small cell neuroendocrine carcinoma). Adenocarcinomas have varying abundances of neuroendocrine cells but it is unknown if adenocarcinomas can be subclassified based on the abundance of neuroendocrine cells (and if so, what is the cutoof?).

2. A recent study has demonstrated that FOXA2 is highly overexpressed in small cell carcinoma and IHC study for FOXA2 is sensitive and specific for small cell carcinoma of the prostate. The current manuscript does not mention this particular molecule in the analysis.

Reviewer #3 (Expertise: Organoids, cancer therapy, Remarks to the Author):

The manuscript is clearly written. The authors have established organoid lines from four CRPC-NE metastasis biopsies; these resemble the original tumor histologically, genetically and transcriptionally. The organoids can be transplanted into mice, and can be used for drug screening. As such, they provide a preclinical model for this tumor type for which, currently, there are not many effective therapeutics available. I do wonder if the story is novel enough, as organoids of this type have been established and published previously (Chen et al, Cell, 2014), although not analyzed as thorougly as they have been done in this work.

I have the following issues:

-In the first part of the manuscript, the authors characterize the established organoid lines. However, they do not specify how long they can culture the organoids, which might be considered as an important characteristic of a new culture system. Moreover, although the authors show that the lines are genetically stable, the way they investigate this is somewhat puzzling. Why would they only check for copy number alterations in specific genes, rather than over the complete genome (which is easily doable and the current gold standard)?

-Another thing that remains unclear is why the authors decide to establish organoids and 2D cell lines, but then don’t investigate the difference. To me it is unclear if -for example- the 2D cell lines can grow for more passages or show different phenotypes in certain analysis that the authors perform. If this is included in the manuscript, it should have a purpose. If both systems work equally well, then why have both?
Just out of curiosity, why do the authors culture the organoids in medium containing DHT, if they actually show that the organoids grow independent of AR signaling? Do they still grow them in the presence of DHT on purpose? And do organoids perhaps change their behavior if this is taken out from the medium? Perhaps this could even be used to validate their claim that neuroendocrine tumors grow in the absence of AR signaling (for example by performing AR staining on organoids cultured with and without DHT)

-Looking at Figure 1, the organoids are mostly characterized by histology. Although the organoids are

certainly positive for neuroendocrine markers such as synaptophysin or chromogranin A, the expression seems to be heterogeneous, and not all cells of the organoids stain positive for this markers. This makes me wonder if the used cultures are completely composed of tumor cells, or if perhaps there is contamination with normal cells (for example liver). As described in earlier work (Chen et al, Cell, 2014), it is possible that these cells over time take over the cultures. This could a.o. be investigated by checking the frequencies of mutations found in the organoid sequencing data. This is currently not included in the manuscript. Another option would be staining with markers for the tissue of which the metastasis was derived (f.e. liver).
-In the experiments described in this manuscript, the authors use both GSK343 and GSK503 to inhibit EZH2. How they decide to use either one of them for different experiments is not clear to me. I'm curious if they have performed all drug screens with both compounds and observed differences between the two compounds or not.

-In my opinion, a crucial missing control in the drugscreens are normal prostate cells, for example healthy prostate organoids. The authors take metastatic adenocarcinoma organoids as a control. Although informative and relevant, I don't think this can replace the normal cell control, as this would give you some initial preclinical information on the therapeutic window of the specific drug treatment.

Point-by-Point Responses to Reviewer Comments

Reviewer #1 (Expertise: Prostate Cancer, Remarks to the Author):

By using an in vitro organoid culture and an in vivo PDX model, the current study recapitulated neuroendocrine prostate cancer from patient specimens, **thoroughly characterized them as valid models** and then utilized these models to evaluate the effects of EZH2 inhibitor in reverting the neuroendocrine tumor back towards an adenocarcinoma phenotype. This **high-throughput organoid drug screening is extremely valuable** in identifying effective single agents and novel drug combinations to treat this **currently undruggable tumor type** in advanced PCa. This approach may provide greater therapeutic opportunities for castration resistant and progressive neuroendocrine prostate cancers and provide a platform for interrogating the molecular underpinnings of adenocarcinoma transdifferentiation towards neuroendocrine tumors in advanced castration-resistant prostate cancer.
Despite the significant strengths of the study, there are multiple issues that need to be addressed.

1. The opening sentence of the paper is inaccurate; PCa is the most common non-cutaneous cancer and the third leading cause of cancer-related deaths in United States men.
Since the time of submission, updated statistics have been reported (Siegel et al., 2018) and prostate cancer is again the second leading cause of cancer-related deaths in men in United States. This reference has been updated in the revised manuscript.

2. Results, first sentence; please make it clear that the 4 organoids were developed from different patients. While stated in the Methods section, it should be clear upon reading the paper without looking that up.
We modified the text in several places including the abstract and results sections to make it clear that the 4 organoids are from 4 different patients.

3. For all of the figures, it is very cumbersome to read through the complicated and inconsistent labeling of the models that include WCM in each label. This was particularly difficult for trying to figure out the meaning of the schematic in Fig 1C, where the tissue is referred to as WCM, the organoid as OWCM, the PDX as PDXWCM, etc. Please re-label this clearly as tissue, organoid, PDX and PDX-derived organoid.
Thank you for the suggestion. We re-labelled all figures as patient biopsy, patient derived organoid (ORG), patient derived organoid xenograft (PDOX), PDOX-organoid to avoid confusion. The revised Fig. 1C is shown below.

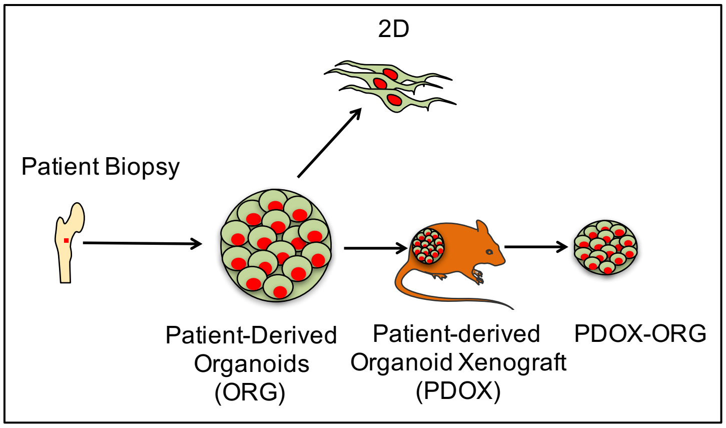

4. Further, the organoid-derived PDX really needs to be labeled and referred to as such rather than simply PDX (e.g. PODX or PDOX). The term PDX conventionally refers to a xenograft derived directly from grafting a patient specimen. While the grafts from organoids may recapitulate some (or all) of the original tumor features, it is critical not to confuse the literature.
We agree and re-labelled them as PDOX in the updated version of the manuscript.

5. Page 6: How does a cytology smear confirm that the cells are tumor?
Cytology was evaluated by a board-certified cytologist at early passages to recognize tumor cells versus normal epithelial cells derived from the tissue where the biopsy had been performed. This method has been described previously in organoids derived from other tumor types (Pauli et al, 2015). Cytology method details and references were added to the revised manuscript.

6. Similarly, a few sentences later, the authors state that the PDXs retained the histologic and morphologic features of the parental tumors. Which features? The histology images are way too small to confirm or refute these vague statements. As a result, the data are not convincing.
We increased the size of the histology images to better appreciate the morphology and immunohistochemistry results. Morphology characteristics were consistent between patient tumor, organoids, and PDOXs in the four patients- each showing small cell carcinoma morphologic features. As shown, all expressed NE markers by IHC and were AR negative.

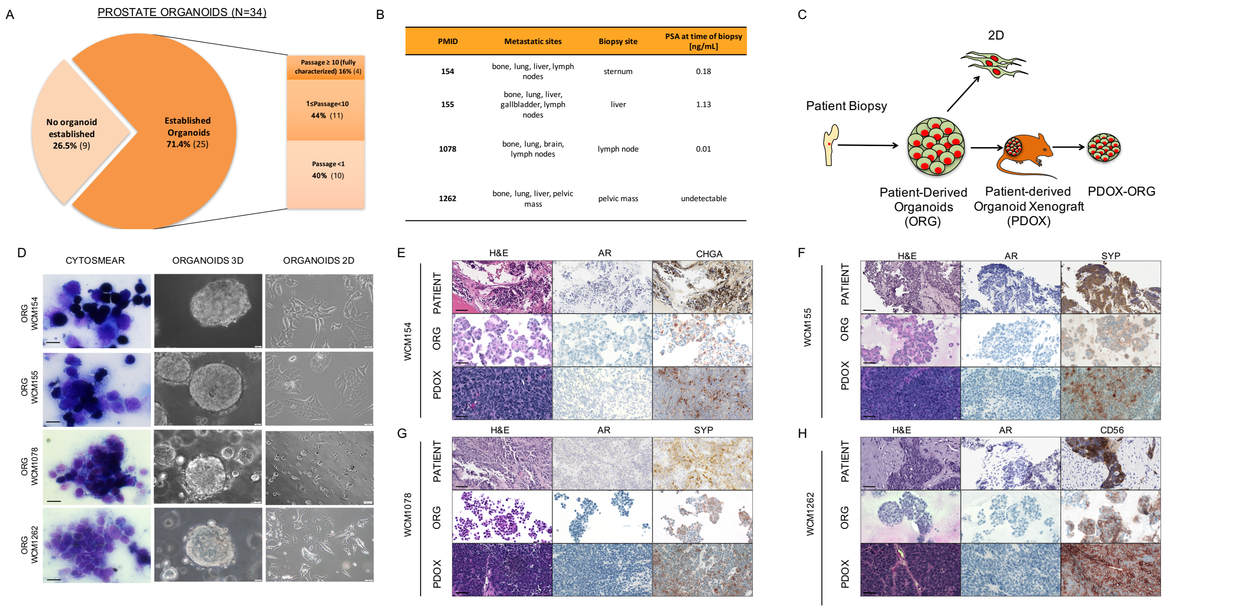

Figure 1

7. The Supplemental figures are presented out-of-order in the paper. Please correct.
The Supplemental Figures are now ordered correctly to reflect the manuscript flow.

8. Page 7: the authors claim that the organoids and PDXs clustered based on their shared expression of gene signatures… However, except for one sample of >20 in Figure 2D, there is no PDX data present.
We have now included data for all four PDOXs and have updated Figure 2B and 2C (below) and all related Supplementary Figures to reflect this.

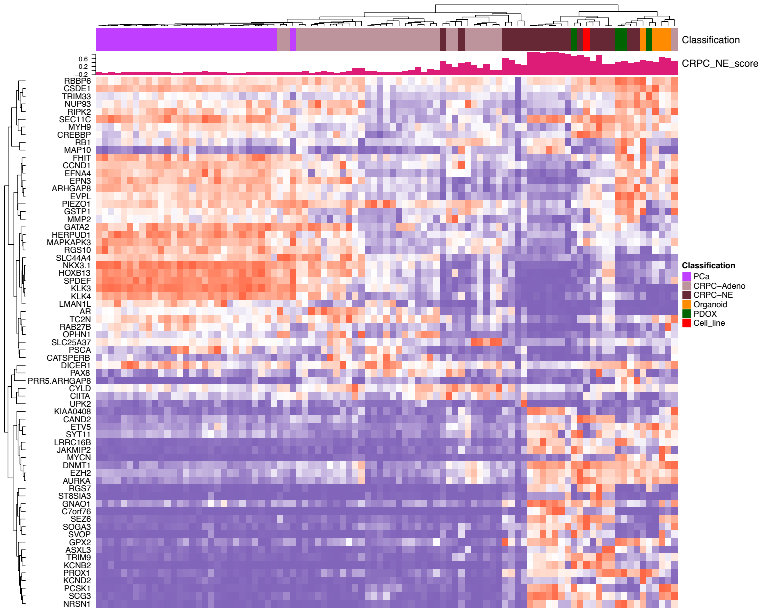

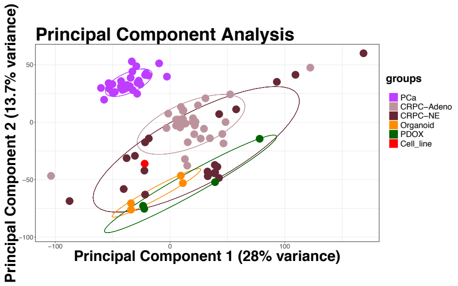

9. Figure 2C: The heat map is labeled as NEPC whereas the term CRPC-NE is used in the text. Consistency is essential with so many models and derivations in this dataset.
We agree that consistency is important. We changed the nomenclature of the Figure to CRPC-NE.

10. Page 8: The authors make a concluding statement that the organoids are clinically relevant systems to further study CRPC-NE pathogenesis. What data supports this statement regarding pathogenesis?
We agree with the Reviewer and we revised the text to reflect this. The main focus of the manuscript focus is on establishing novel models and exploring therapies.

11. The EZH2 inhibitor GSK343 treatment resulted in a preferential decrease in the viability of CRPC-NE organoids. EZH2 knockdown by shRNA decreased the expression of classical CRPC-NE markers. Did EZH2 knockdown by shRNA result in a similar effect on organoids growth as treatment with the EZH2 inhibitor?
We observed that CRPC-NE organoids die over time in culture after infection with shEZH2 suggesting a dependency on the presence of EZH2 for their growth. However, we were able to measure the effect of shEZH2 on proliferation in the first week of infection and compare with GSK343 treatment (IC50 used) using Incucyte. As shown below, we observed a reduction in proliferation of organoids after shEZH2 similar to that seen in organoids after GSK343 treatment.

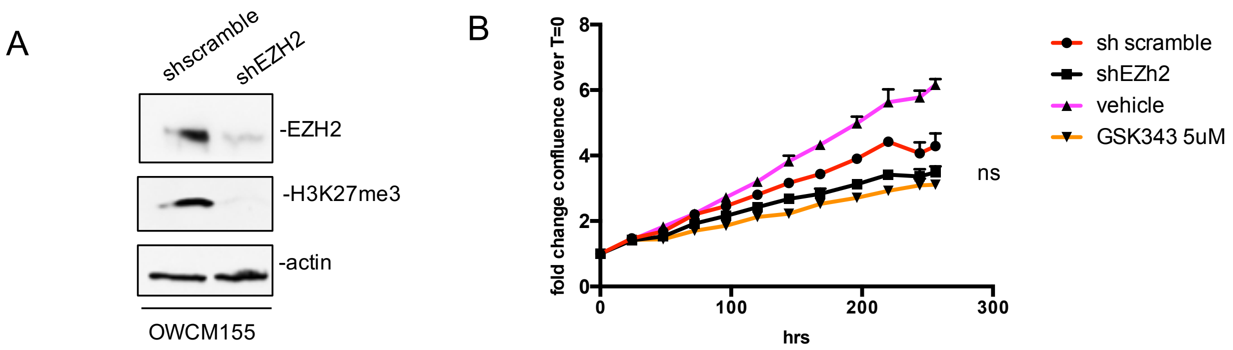

12. To increase the clinical relevance of this methodologic paper, the in vivo PDX model should be employed to test the effects of EZH2 inhibitor and other single or combination therapies.

We appreciate this comment. To address this, we performed a pilot EZH2 inhibitor experiment in vivo using PDOXs (data shown below). We observed a modest reduction of tumor growth, but EZH2 inhibition alone was insufficient to arrest tumor growth. This is similar to what has been reported by others in prostate cancer (Ku et al., 2017) showing that single agent activity is limited. However, EZH2 inhibition resulted in changes in the expression of neuroendocrine markers (such as synaptophysin) both in vivo and in vitro (shown in Figure 3A). These results provide further rationale for exploring EZH2 drug combinations as we pursued with the drug screen.

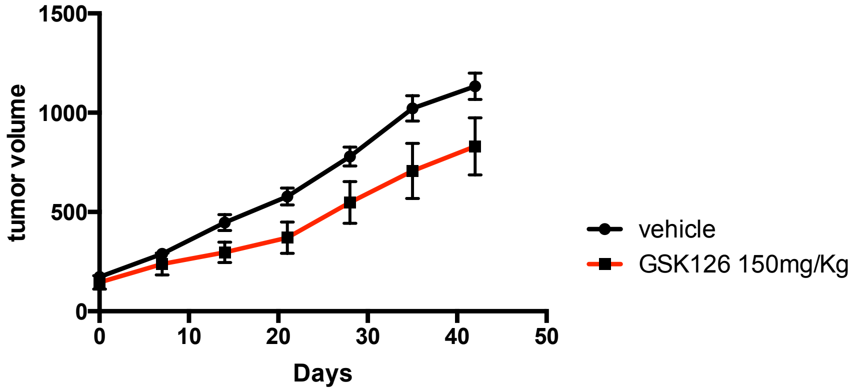

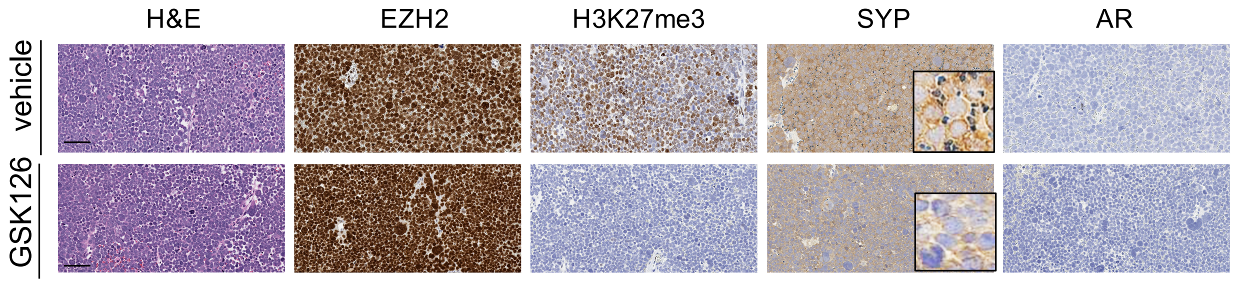

13. Page 24 under Methods: For each PDX graft, “1.5 million” of organoids were injected sounds impossible. Do the authors mean 1.5 million cells from organoid cultures? How long do the PDXs

grafts grow under SQ? Are NOD SCID mice males or females? Age? Animal protocol? Housing? These need to be described in more detail.
We injected 1.5 million of cells derived from organoids in NOD scid gamma (NSG) male mice in 1:1 mix with Matrigel (average 6-8 weeks old). The time for engraftment growth varied in each patient derived organoid xenograft with an average of 3 months. The growth was arrested when the tumor reached the limit established by IACUC guidelines (tumor volume =2cm^3). We included details in the methodology section of the manuscript as suggested.

14. Dr. Vessella's group has developed many prostate cancer patient-derived xenograft lines including neuroendocrine cancer line LuCaP49 with intensive publications. This work needs to be discussed.
Thank you for the suggestion. We have included the LuCaP49 model in the Discussion session of the manuscript.

15. There are some typos and grammar issues scattered throughout that the authors need to better edit.
We edited the updated manuscript to correct grammar and typos.

Reviewer #2 (Expertise: Prostate cancer genomics, Remarks to the Author):

In the manuscript by Puca et al, the authors report their work in the development and characterization of patient derived organoids to model small cell prostate cancer. A few such models were successfully established, and the authors demonstrate genomic, transcriptomic and epigenomic concordance between organoids and their corresponding patient tumors. Finally, the authors utilize these organoids to understand the biologic role of the epigenetic modifier EZH2 in driving molecular programs associated with lineage plasticity and neuroendocrine progression. High-throughput organoid drug screening identified single agents and novel drug combinations suggesting repurposing opportunities.

Although patient-derived organoids have been used in the study of many cancers including prostate cancer, **having small cell prostate cancer model is highly valuable**. In this regard, this group, which has been leading prostate cancer genomics studies, has made **important new contributions to the field**. The EZH2 study is less novel. **However, it does help to validate the models** and gives us an opportunity to look at the potential of using EZH2 inhibitor for the treatment of small cell prostate carcinoma. **The high-throughput drug screening represents an important step** to introduce the obtained model resources into translational utility.

Therefore, **despite some weakness in novelty, I still believe this represents strong work that will lead us a step closer towards better understanding and potential therapeutic strategy of small cell carcinoma.** I have a few suggestions for the authors:

1. Terminology: The names used to describe the tumor pathology are somewhat inconsistent. Throughout the manuscript, the authors used "neuroendocrine prostate cancer", " small cell neuroendocrine

carcinoma", "high-grade carcinoma with extensive neuroendocrine differentiation". This is confusing to readers. Do they all mean the same tumor or do they have different meanings? I understand there is a historic reason for this confusion, and a recent paper (reference 9) failed to adequately address this. However, the authors have a responsibility to use a consistent term if they are talking about the same tumors because to pathologists, there are adenocarcinomas and small cell carcinomas (or small cell neuroendocrine carcinoma). Adenocarcinomas have varying abundances of neuroendocrine cells but it is unknown if adenocarcinomas can be subclassified based on the abundance of neuroendocrine cells (and if so, what is the cutoof?).

We thank the Reviewer for these important comments. We have revised the manuscript nomenclature to be consistent throughout. Neuroendocrine prostate cancer is typically defined as either pure small cell carcinoma or prostate adenocarcinoma with extensive small cell/neuroendocrine differentiation (Epstein et al, 2014). These exact cutoffs have not been established clinically yet. Metastatic biopsies have only recently been performed in the setting of treatment resistant prostate cancer and emerging data from the two Stand up to Cancer-Prostate Cancer Foundation Dream Teams (>500 cases, unpublished) will help define these cutoffs better. As collaborators on the two SU2C-PCF dream teams, the frequency of neuroendocrine prostate cancer in metastatic biopsies is up to 20% in both the pre-abiraterone/enzalutamide and post-therapy CRPC setting (similar frequency as a recent rapid autopsy study by Bluemn et al Cancer Cell 2017). These data suggest that the landscape of advanced prostate cancer is only recently evolving and the clinical/pathologic/molecular criteria of defining neuroendocrine prostate cancer will also continue to evolve. Having said that, the models described here are predominantly small cell carcinoma, AR negative, and express NE markers. This was now emphasized in the text.

2. A recent study has demonstrated that FOXA2 is highly overexpressed in small cell carcinoma and IHC study for FOXA2 is sensitive and specific for small cell carcinoma of the prostate. The current manuscript does not mention this particular molecule in the analysis.
We agree with the Reviewer. FOXA2 is also overexpressed in our internal CRPC-NE patient cohorts and CRPC-NE models (shown below). We added these data as a new Supplementary Figure S5 that also includes relevant CRPC-NE genes for comparison.

FOXA2

Expression values in Prostate samples

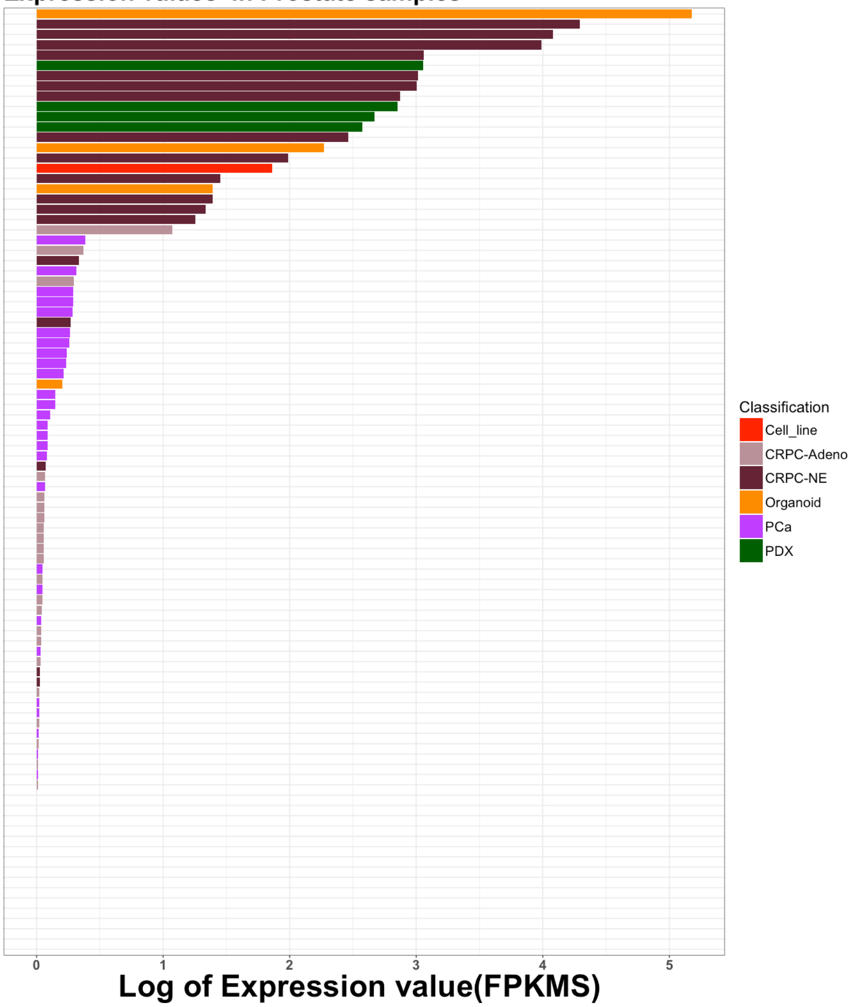

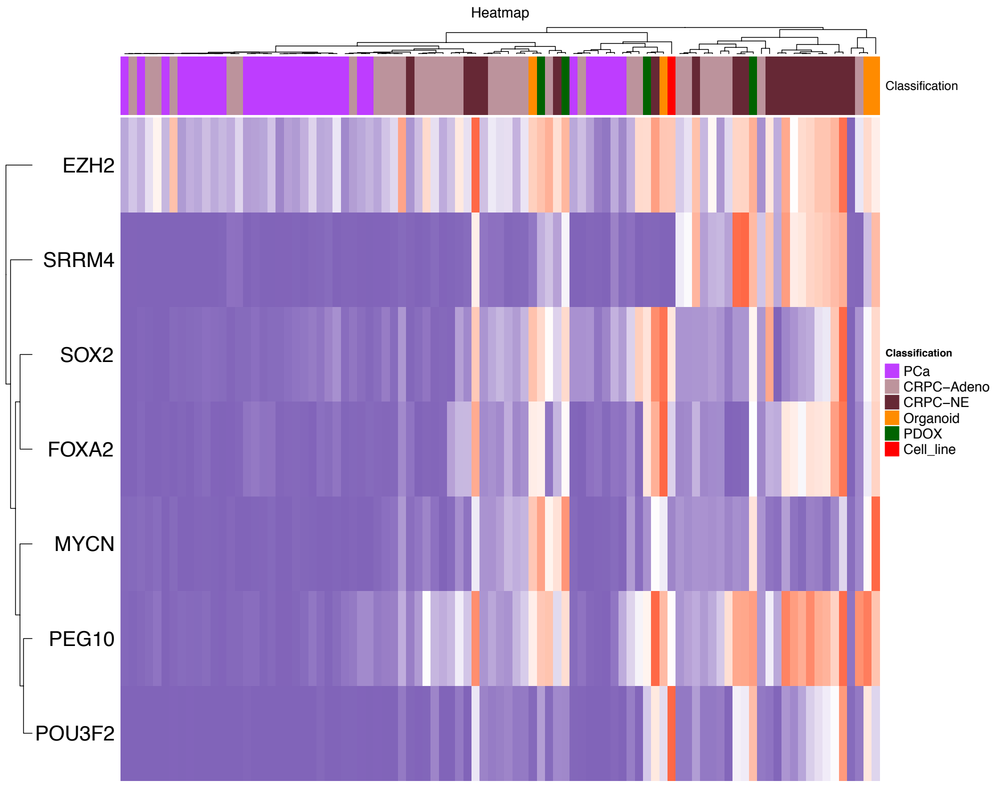

Reviewer #3 (Expertise: Organoids, cancer therapy, Remarks to the Author):

The manuscript is clearly written. The authors have established organoid lines from four CRPC-NE metastasis biopsies; these resemble the original tumor histologically, genetically and transcriptionally. The organoids can be transplanted into mice, and can be used for drug screening. **As such, they provide a preclinical model for this tumor type for which, currently, there are not many effective therapeutics available**. I do wonder if the story is novel enough, as organoids of this type have been established and published previously (Chen et al, Cell, 2014), although not analyzed as thoroughly as they have been done in this work.

I have the following issues:

-In the first part of the manuscript, the authors characterize the established organoid lines. However, they do not specify how long they can culture the organoids, which might be considered as an important characteristic of a new culture system.

The established organoids can be continually expanded. We were able to culture them over 30 passages in culture, freeze and thaw them, and continuously expand them again over time in culture (now over 3 years). A limiting factor in the field and also contributing to the low success rate of prostate organoids is the risk of senescence after a few passages in culture that affects the ability to manipulate them for downstream studies. In Gao et al, 2014, we established CRPC

organoids including one derived from a pleural effusion that had neuroendocrine features. However, keeping this neuroendocrine organoid from Gao et al in culture and performing downstream analyses has been challenging, and consistent with the difficulty of the field of having functional models for neuroendocrine prostate cancer. The novelty of our work stands in the fact that we established both organoids and 2 dimensional lines that may be indefinitely expanded and used to generate hypotheses for treatment and for gene manipulation. These data and areas of novelty are highlighted in the revised manuscript.

Moreover, although the authors show that the lines are genetically stable, the way they investigate this is somewhat puzzling. Why would they only check for copy number alterations in specific genes, rather than over the complete genome (which is easily doable and the current gold standard)?

Based on this suggestion, we have added a new figure (as shown below, now as Supplementary Figure S4) showing genome-wide genomic alterations observed in matched tissue, organoids and corresponding xenografts. It is evident in the plot that WCM154 and WCM155 demonstrate whole genome duplication which is retained across sample types, while WCM1078 and WCM1262 do not. Genomic alterations were conserved along the whole exome between tissue and organoids and PDOXs in all 4 cases. In case WCM1078, there was one area of the genome (at chromosome 8; a region typically altered in prostate cancer) that may have been selected for in late passage organoids and PDOX models due to subclonal selection. Overall these data suggest that patients organoids and PDOXs are representative of their matched tumor at a genome wide scale.

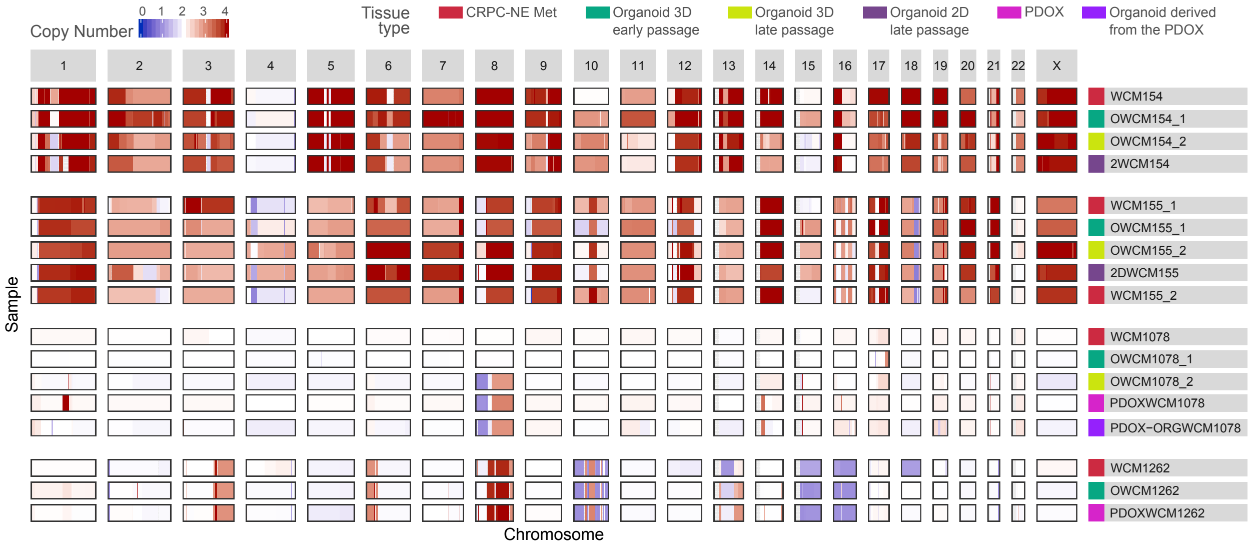

-Another thing that remains unclear is why the authors decide to establish organoids and 2D cell lines, but then don't investigate the difference. To me it is unclear if -for example- the 2D cell lines can grow for more passages or show different phenotypes in certain analysis that the authors perform. If this is included in the manuscript, it should have a purpose. If both systems work equally well, then why have both?

We established both 3D and 2D cell lines in order to understand whether 2D may be used as additional models as they require less resources and technical processing compared with 3D organoids for downstream functional studies. As shown in the DNA copy number figure, genomic alterations are retained across both 3D and 2D organoids. For the revision, we are also providing an additional figure (Supplementary Figure S7) that demonstrates concordant gene expression (RNA-seq) between the two systems as well as representative western blots.

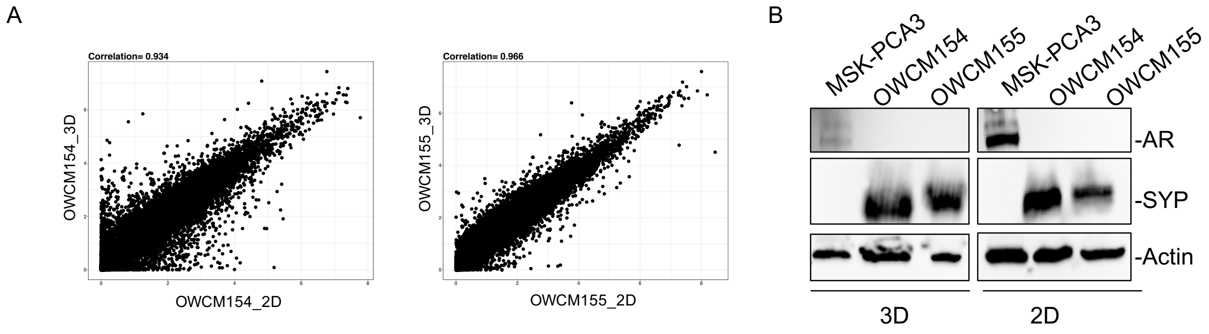

Just out of curiosity, why do the authors culture the organoids in medium containing DHT, if they actually show that the organoids grow independent of AR signaling? Do they still grow them in the presence of DHT on purpose? And do organoids perhaps change their behavior if this is taken out from the medium? Perhaps this could even be used to validate their claim that neuroendocrine tumors grow in the absence of AR signaling (for example by performing AR staining on organoids cultured with and without DHT)

We appreciate Reviewer's comment. We usually culture organoids in DHT conditions since at the moment we process the biopsy to try and maintain the heterogeneity that we often observe in patients with mixed tumors without selecting clones that do not need DHT to survive. As suggested, we removed DHT from the culture media for 24 hrs and 7 days, extracted mRNA and evaluated the expression of AR signaling genes and neuroendocrine markers (Figure below). There was overall similar profiles with or without DHT and no observed change in behavior. This data was added to the revised manuscript as Supplementary Figure S8.

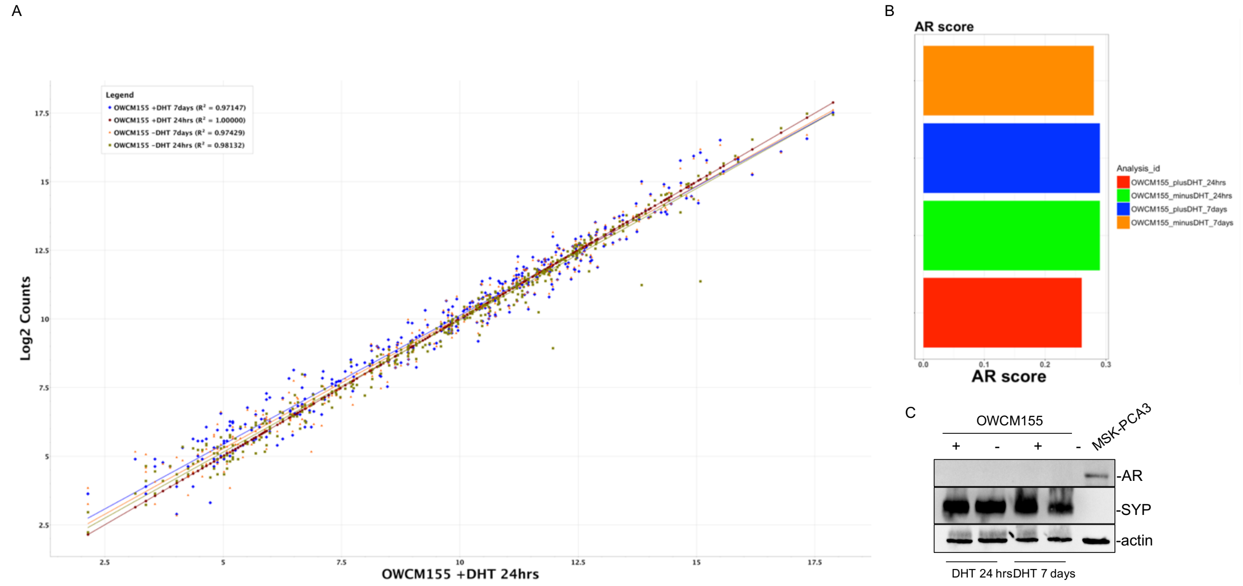

-Looking at Figure 1, the organoids are mostly characterized by histology. Although the organoids are certainly positive for neuroendocrine markers such as synaptophysin or chromogranin A, the expression seems to be heterogeneous, and not all cells of the organoids stain positive for this markers. This makes me wonder if the used cultures are completely composed of tumor cells, or if perhaps there is contamination with normal cells (for example liver). As described in earlier work (Chen et al, Cell, 2014), it is possible that these cells over time take over the cultures. This could a.o. be investigated by checking the frequencies of mutations found in the organoid sequencing data. This is currently not included in the manuscript. Another option would be staining with markers for the tissue of which the metastasis was derived (f.e. liver).

We appreciate the suggestion and have now added corresponding data of tumor organoid and PDOX tumor purity as well as genomic and tissue specific marker evaluation. The tumor purity was high in organoids and PDOX models (median 98%). The copy number and mutational profiles also supported highly clonal tumor specific alterations. We also performed IHC and Western Blot of PTEN in organoids (as shown below) and confirmed PTEN homozygous loss compared to a CRPC-NE organoids with PTEN wild type. As suggested by the Reviewer, we performed IHC to evaluate Hep Par1 expression as a well described liver marker in the liver metastasis-derived organoid. We did not observe expression in the organoid culture (positive control benign liver is shown in the inset) suggesting that the organoid culture is entirely represented by tumor cells and the heterogeneity observed with neuroendocrine markers by IHC is the heterogeneity that we generally observe in patients as well. These results are also reported in Supplementary Figures S3, S10, S11.

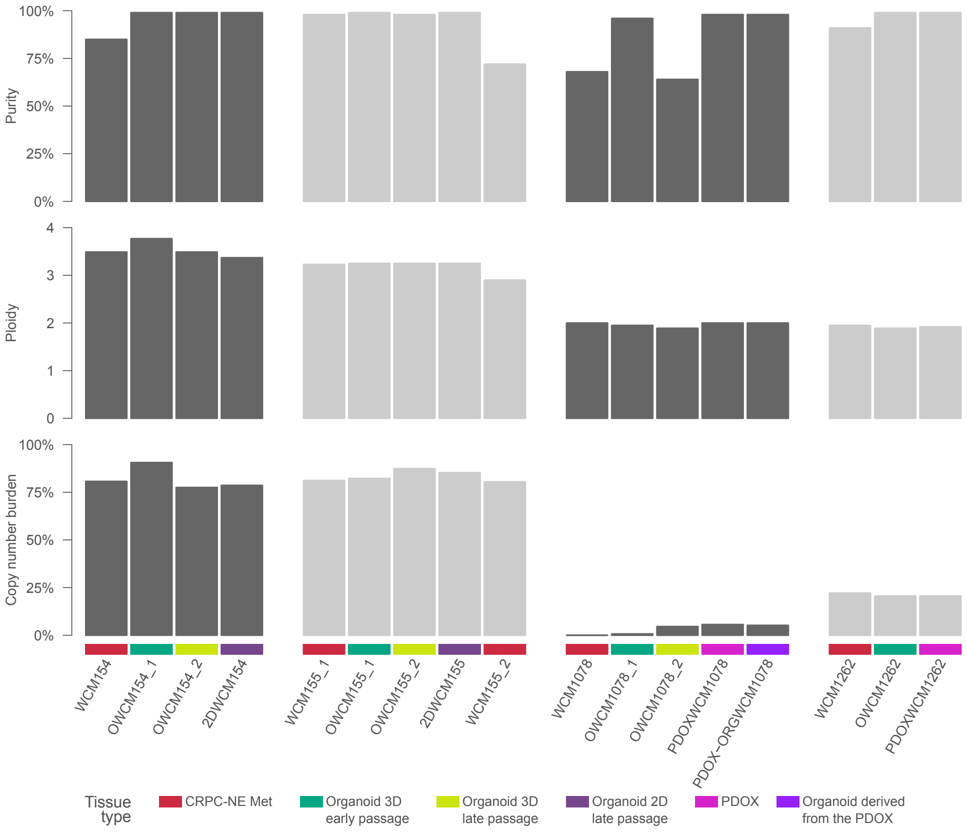
Purity
100%
75%
50%
25%
0%
Ploidy
4
3
2
1
0
Copy number burden
100%
75%
50%
25%
0%
WCM154
OWCM154_1
OWCM154_2
2DWCM154
WCM155_1
OWCM155_1
OWCM155_2
2DWCM155
WCM155_2
WCM1078
OWCM1078_1
OWCM1078_2
PDOXWCM1078
PDOX−ORGWCM1078
WCM1262
OWCM1262
PDOXWCM1262
Tissue type
CRPC-NE Met
Organoid 3D early passage
Organoid 3D late passage
Organoid 2D late passage
PDOX
Organoid derived from the PDOX

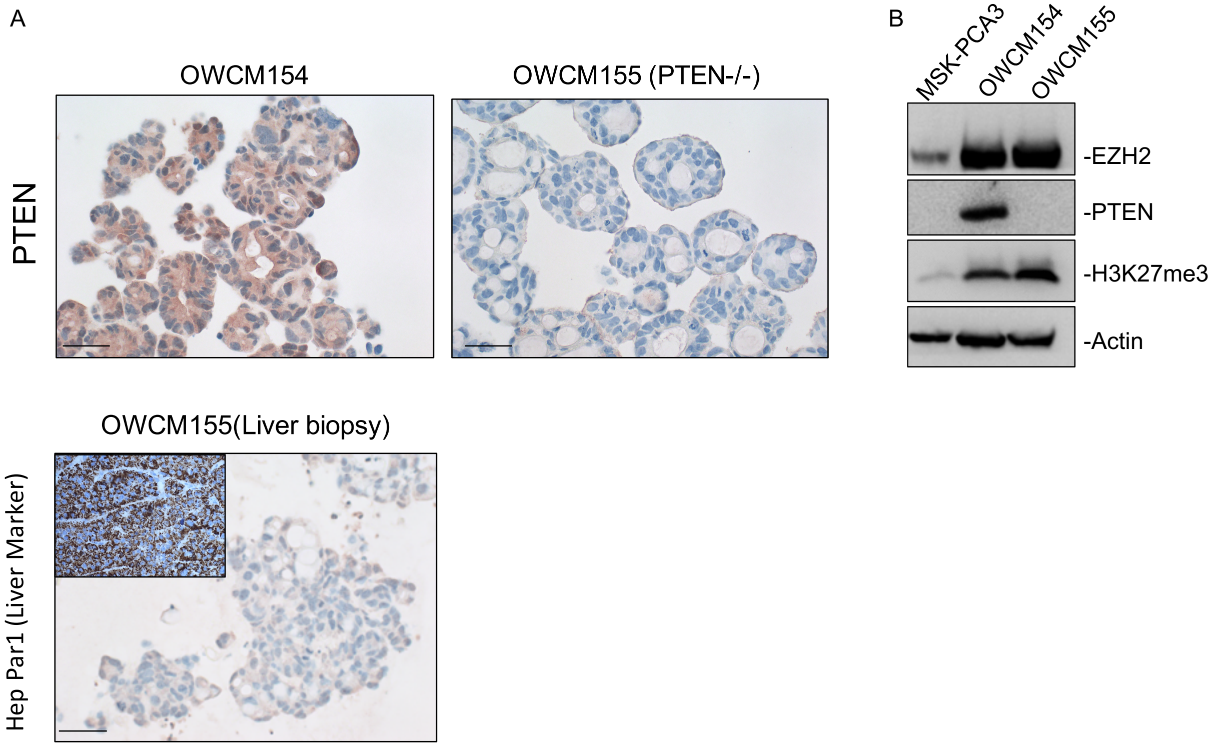
A
OWCM154
OWCM155 (PTEN-/-)
PTEN
OWCM155(Liver biopsy)
Hep Par1 (Liver Marker)
B
MSK-PCA3
OWCM154
OWCM155
-EZH2
-PTEN
-H3K27me3
-Actin

-In the experiments described in this manuscript, the authors use both GSK343and GSK503 to inhibit EZH2. How they decide to use either one of them for different experiments is not clear to me. I'm curious if they have performed all drug screens with both compounds and observed differences between the two compounds or not.

Based on discussions with GSK and previous publications (Lian Dee Ler et al.,2017, Beguelin et la., 2013), GSK343 and GSK503 have a similar effect on H3K27me3, as we now also show in Supplementary Figure S15 and report here. We performed a cell viability assay where we see that both compounds have comparable effects on the organoids. For the drug screening, we decided to use GSK503 since it has a better pharmacokinetics in vivo, as communicated by GSK company, therefore results can be easily translated in vivo for future studies. H3K27me3 expression results are also reported in Supplementary Figure S15.

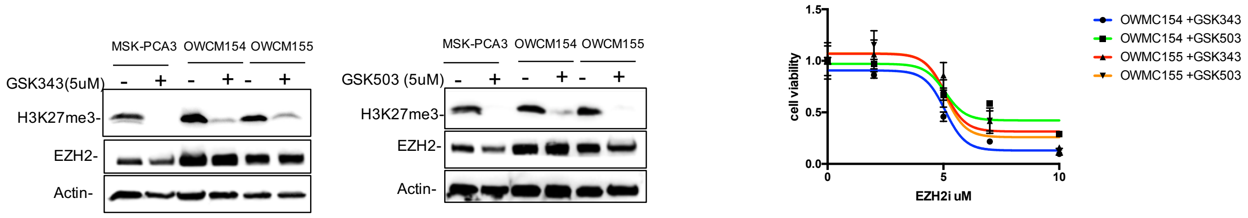

-In my opinion, a crucial missing control in the drugs screens are normal prostate cells, for example healthy prostate organoids. The authors take metastatic adenocarcinoma organoids as a control. Although informative and relevant, I don't think this can replace the normal cell control, as this would give you some initial preclinical information on the therapeutic window of the specific drug treatment.

We thank the Reviewer for this suggestion. We performed validation of the compounds highlighted in the manuscript (GSK343 and alisertib) using both cancer (CRPC-Adeno, CRPC-NE) and control benign prostate cells, RWPE-1 cell line, as well as a patient-derived benign prostate organoid model (WCM1420 ORG). As shown below, benign cells (both RWPE and WCM1420 ORG) are insensitive to treatment with GSK343. RWPE were sensitive to alisertib but only at high doses (OWCM155 IC50=55.15nM, RWPE IC50= 6825nM) and benign organoids were completely insensitive. These data are now included as Supplementary Figure S19.

A

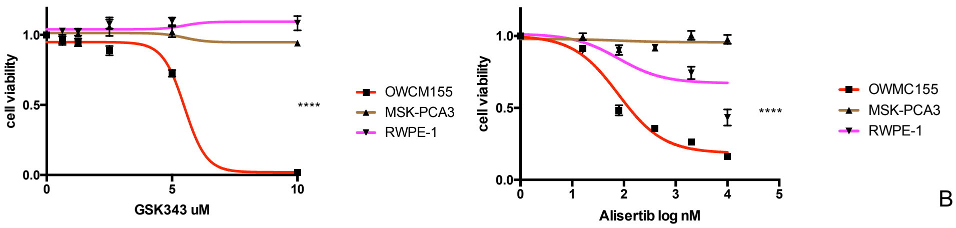

cell viability
1.0
0.5
0.0
0
5
10
GSK343 uM
****
OWCM155
MSK-PCA3
RWPE-1
cell viability
1.0
0.5
0.0
0
1
2
3
4
5
Alisertib log nM
****
OWMC155
MSK-PCA3
RWPE-1

B

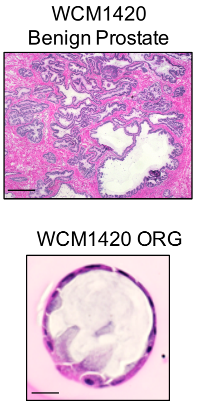

WCM1420
Benign Prostate
WCM1420 ORG

C

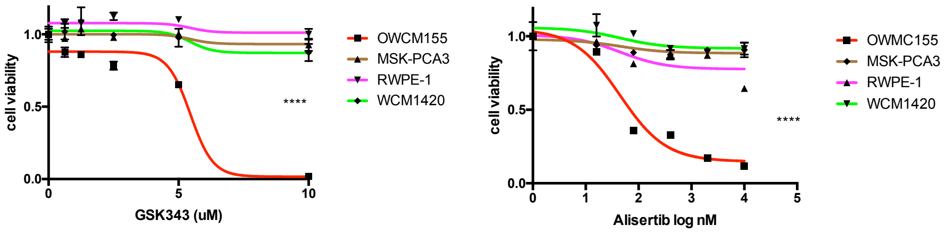

cell viability
1.0
0.5
0.0
0
5
10
GSK343 (uM)
****
OWCM155
MSK-PCA3
RWPE-1
WCM1420
cell viability
1.0
0.5
0.0
0
1
2
3
4
5
Alisertib log nM
****
OWMC155
MSK-PCA3
RWPE-1
WCM1420

REVIEWERS' COMMENTS:

Reviewer #1 (Remarks to the Author):

The previous concerns have been fully addressed with new data and adequate adjustments to the manuscript. There are no further concerns.

Reviewer #2 (Remarks to the Author):

The revised manuscript has addressed all the concerns raised by the previous reviews. It is acceptable for publication in Nature Communications.

Reviewer #3 (Remarks to the Author):

the authors have addressed the questions raised clearly in my opinion. Therefore I propose to accept the revised manuscript.
